# Magnetization Switching of Single Magnetite Nanoparticles Monitored Optically

**DOI:** 10.1021/acs.nanolett.4c01850

**Published:** 2024-07-30

**Authors:** Subhasis Adhikari, Yonghui Wang, Patrick Spaeth, Francesca Scalerandi, Wiebke Albrecht, Junyan Liu, Michel Orrit

**Affiliations:** † Huygens-Kamerlingh Onnes Laboratory, Leiden University; 2300 RA Leiden, The Netherlands; ‡ School of Mechatronics Engineering, Harbin Institute of Technology; Harbin 150001, People’s Republic of China; § Department of Sustainable Energy Materials, AMOLF; Science Park 104, 1098 XG Amsterdam, The Netherlands

**Keywords:** photothermal circular dichroism microscopy, magnetic circular dichroism, single-particle spectroscopy, dynamical heterogeneity, magneto-optical Kerr effect, magnetic nanomaterials

## Abstract

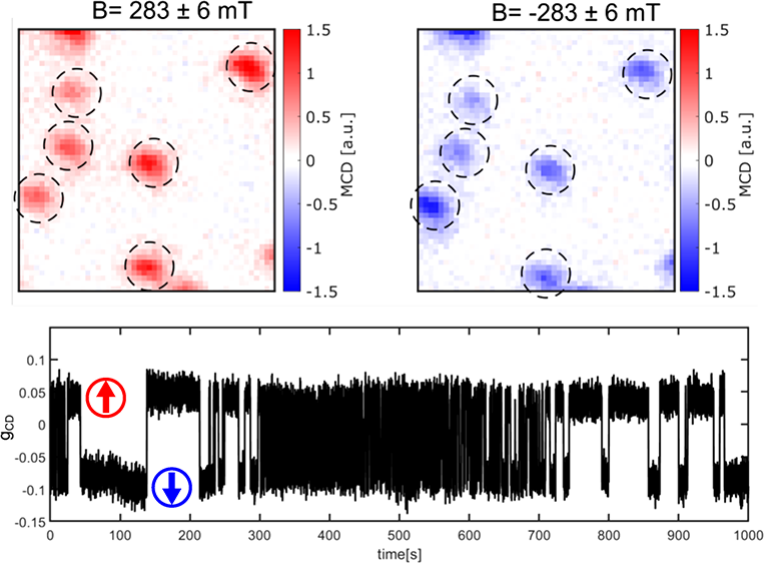

Magnetic nanomaterials record information as fast as picoseconds in computer memories but retain it for millions of years in ancient rocks. This exceedingly broad range of times is covered by hopping over a potential energy barrier through temperature, ultrafast optical excitation, mechanical stress, or microwaves. As switching depends on nanoparticle size, shape, orientation, and material properties, only single-nanoparticle studies can eliminate the ensemble heterogeneity. Here, we push the sensitivity of photothermal magnetic circular dichroism down to *individual* 20 nm magnetite nanoparticles. Single-particle magnetization curves display superparamagnetic to ferromagnetic behaviors, depending on the size, shape, and orientation. Some nanoparticles undergo thermally activated switching on time scales of milliseconds to minutes. Surprisingly, the switching barrier varies with time, leading to dynamical heterogeneity, a phenomenon familiar in protein dynamics and supercooled liquids. Our observations will help to identify the external parameters influencing magnetization switching and, eventually, to control it, an important step for many applications.

Magnetic nanomaterials,^1−7^ including nanoparticles, promise numerous applications in fields as varied as nanotechnology for data storage, sensing and logics,^8^ geomagnetism,^9^ magnetothermal therapy in medicine,^10^ and the biomagnetic compass of bacteria and birds.^11^ In all those fields of application, however, the heterogeneity of magnetic nanomaterials is an obstacle to a better characterization and understanding of their magnetic properties. Single-nanoparticle studies^12^ are required to overcome ensemble averaging and open the correlation of magnetic properties with nanoparticle composition, size, shape,^13,14^ orientation, and structure.^15^ Several techniques can reach single-nanoparticle magnetization sensitivity, from electrical current measurements^16^ to scanning probe microscopies.^17−20^ Those techniques, however, are complex and often require contacts and/or scanning probes, which may alter the sample’s magnetic properties. Noncontact optical techniques are thus particularly attractive. Setting aside X-ray MCD (XMCD) measurements at synchrotrons,^14^ conventional optical Kerr microscopy based on the magneto-optical Kerr effect (MOKE) lacks the spatial resolution needed to address single nanoparticles, with the notable exception of magnetometry with NV-centers in diamond.^21^ We recently proposed an original optical technique, photothermal magnetic circular dichroism (PT MCD) microscopy,^22^ which has the potential to optically record (time-resolved) magnetic properties of single magnetic nanoparticles (see basic principles of PT MCD in the Supporting Information). The key advantages of PT MCD over other single-particle methods are as follows. (i) PT MCD is simpler in design and cheaper. It only requires a tabletop microscope in a small-scale lab. (ii) The sample can be reused after several treatments, providing information about parameters influencing its magnetic properties. In this work, we improved our optical setup by reducing the heating beam’s area and by improving our control of its polarization. Thereby, we demonstrate the experimental imaging of single 20 nm magnetite nanoparticles, and we record their full magnetization curves, one particle at a time. In this method, the single-particle (polar) MOKE signal, which gives rise to a slight magnetic-field-induced difference in optical absorption for right- and left-circularly polarized light, is detected by the scattering of a tightly focused probe beam. The resulting magnetization curves hold information about the magnetic properties of the particles. In small enough particles of a ferromagnetic (or ferrimagnetic in the case of magnetite) material, all spins are aligned by exchange interactions. Such particles present a single magnetic domain. Their total magnetic moment or macro-spin, however, can still switch as a whole between different orientations under thermal fluctuations. Whenever the switching is much slower than the characteristic measurement times, the magnetization appears to be frozen. For switching much faster than measurements, the particles are said to be superparamagnetic because they behave as a paramagnetic species with a large magnetic moment. In particular, their average magnetization under zero applied magnetic field is nil.^23,24^ Note that the definition of superparamagnetism depends on the experimental time resolution and temperature. The temperature above which a particle becomes superparamagnetic (for a given time resolution, typically seconds) is called the blocking temperature. In comparison to our previous PT MCD measurements of magnetite nanoparticulate clusters, about 400 nm in size, we herein study isolated single nanoparticles with volumes about 4 orders of magnitude smaller, small enough to present a single magnetic domain. In addition, we demonstrate thermally activated switching between two antiparallel magnetization states, and we visualize switching time traces of up to hours with a time resolution as high as 10 ms. Magnetic switching, predicted by Néel some 70 years ago, can now be followed by our technique in real time on single magnetite particles, the type of particles that are thought to have recorded paleomagnetic data in ancient rocks. Magnetization curves provide us with estimates of the shape anisotropy and easy-axis orientation of each single nanoparticle according to the Stoner−Wohlfarth model. A recent article^25^ has applied the Stoner−Wohlfarth model in a similar way to determine the magnetic anisotropy constant and the easy-axis orientation of 15 single magnetite nanoparticles in a bacterium using XMCD. These particles, however, were larger (∼50 nm) than ours and, instead of our table-top optical setup, the X-ray microscopy required a synchrotron facility. By varying the applied magnetic field and temperature, we deduce the particles’ magnetic dipoles, around 10^5^ μ_B_, and the switching barrier’s activation energies, around 0.8 eV. Long switching time traces display pronounced changes in switching rate, i.e., dynamical heterogeneity, indicating that the barrier can fluctuate significantly with time. Such dynamical heterogeneity is well-known in the dynamics of proteins and of supercooled liquids but had not been reported previously for magnetic nanoparticles.

Figure 1A shows a photothermal (PT) image of six single magnetite nanoparticles, labeled P1–P6, with average diameters ranging from about 19 to 25 nm. The sizes, mentioned in the inset in Figure 1E–J, are deduced from a comparison of the histogram of photothermal signals of a large number of such nanoparticles (see Figure S1) to their average diameter, about 19 nm, obtained from transmission electron microscope (TEM) images of 38 particles (see Figure S2) and assuming a linear relationship between a particle’s photothermal signal and its volume (see details about size estimation in the Supporting Information). A histogram of signal-to-background ratios of 465 single magnetite nanoparticles is shown in Figure S1, with a mean signal-to-background (S/B) ratio of about 40. Such a high visibility indicates that even smaller magnetite nanoparticles could be detected with our photothermal setup.

**Figure 1:**
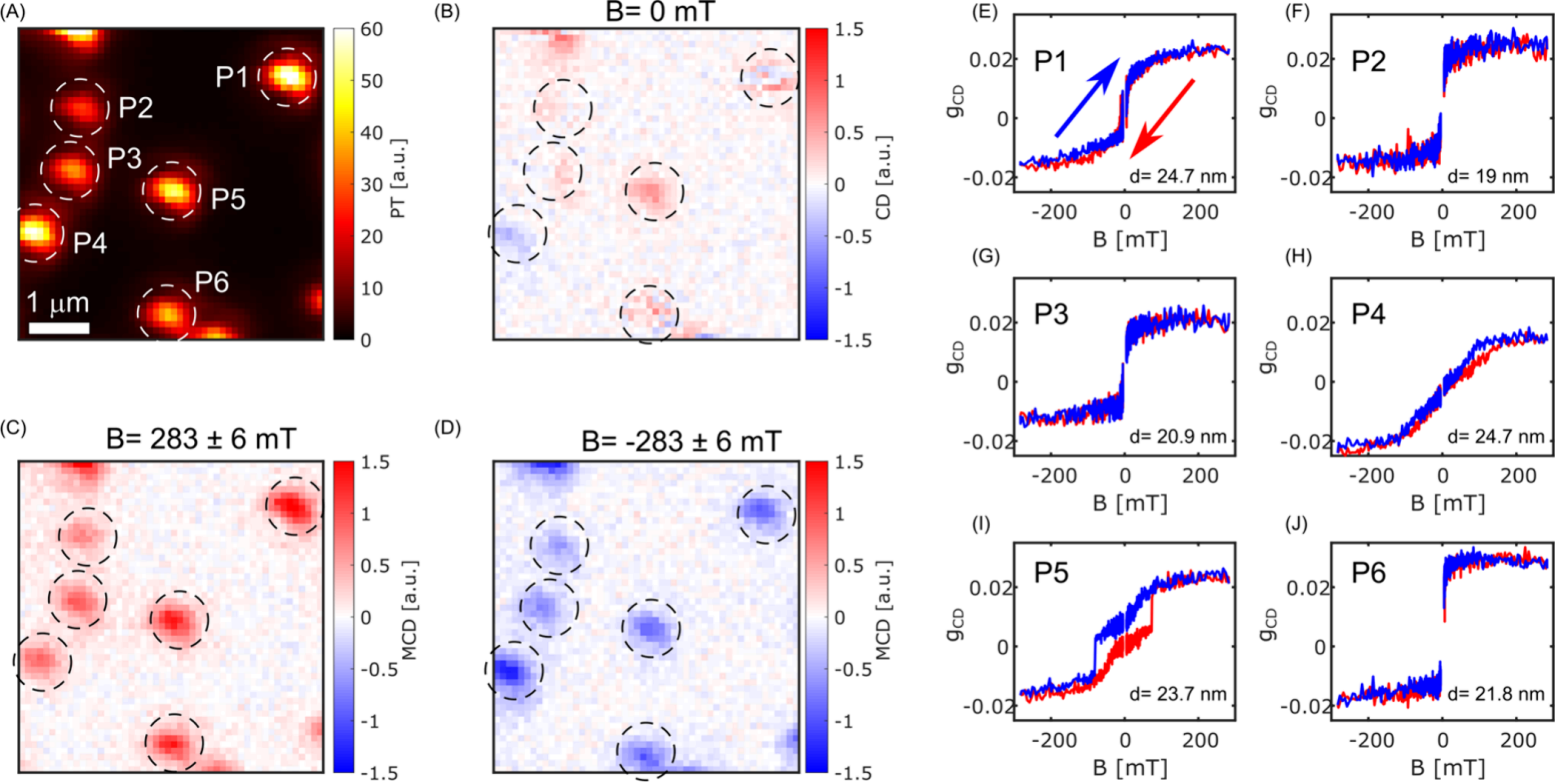
Photothermal CD images of single magnetite nanoparticles about 20 nm in diameter. (A) Photothermal (PT) image. (B) CD image without applied static magnetic field. (C, D) MCD images with magnetic field applied along the microscope’s optical axis (±283 mT, respectively). The signal units of images (A–D) are mutually consistent. The background in (C) and (D) also appears to flip sign with the magnetic field orientation. We assign it to a weak MCD signal from the permanent magnet when it is placed close to the sample. (E–J) Dependence of the dissymmetry factor *g*_CD_ on magnetic field for the six nanoparticles P1–P6. The indicated average diameters of the particles are deduced from their PT signal. Particle P1 shows switching between positive and negative dissymmetry factors *g*_CD_ at weak fields, whereas particle P5 shows hysteresis. The colors indicate the scan direction of the applied field, as indicated in (E). Some of these magnetization curves are not (anti)symmetric around zero field (see nanoparticles P2, P3, and P6). We assign these shifts to weak geometrical chirality of these nanoparticles.

By modulating the heating beam between right- and left-handed circular polarizations, we observe the circular dichroism (CD) response of particles P1–P6, first in the absence of a magnetic field (Figure 1B). We assign most of the weak signals observed to geometric CD stemming from a low dielectric polarizability of magnetite at our pump wavelength of 532 nm, and from non-mirror-symmetrical (chiral) particle shapes. The significant and consistent positive signal of particle P5, however, suggests a possible ferromagnetic behavior. Upon application of a static magnetic field of ±(283 ± 6) mT along the microscope’s optical axis, all particles acquire strong CD signals, which change sign with the field direction (Figure 1C,D), indicating magnetic circular dichroism (MCD). The signal-to-noise (S/N) ratio exceeds 10 for an integration time of 100 ms/pixel, demonstrating the high sensitivity of the technique (note that we use S/N instead of S/B for the MCD signal because the MCD background fluctuates around zero). The saturation magnetic moment expected for particle P1 can be deduced from its volume and from the side of the cubic unit cell of magnetite, 0.839 nm. With 32 Bohr magnetons per unit cell,^26^ we expect a magnetic moment of 4.4 × 10^5^ μ_B_ at saturation. The detection sensitivity of our method is thus better than 4.4 × 10^4^ μ_B_.

Our MCD measurements enable us to record the full magnetization curves of single magnetite particles. From the magneto-optical signal MCD = *I*_–_ – *I*_+_, i.e., the difference in circularly left (*I*_–_)- and right-polarized (*I*_+_) absorption, and from the unpolarized photothermal absorption, PT = (*I*_–_ + *I*_+_)/2, we deduce the dissymmetry factor, . The magnetization curves of particles P1–P6 in Figure 1E–J show that the magnetic properties of individual particles are strikingly distinct. According to the Néel–Brown model,^23^ superparamagnetism is observed when the magnetization switches much faster than the measurement time, so that the net magnetization is zero without any external field, whereas ferromagnetism is observed when the magnetization switching is much slower than the measurement time and there is a hysteresis. Particles P2–P4 and P6 display superparamagnetic behavior, with a regular increase of magnetization with applied field. In contrast, particle P5 shows a typical ferromagnetic behavior indicated by a clear hysteresis loop and a coercive field of about 100 mT. Superparamagnetic particles P2, P3, and P6 show saturation at much lower fields than particle P4. We attribute this difference to the orientation of their magnetic easy axis, which is presumably nearly aligned with the field for particles P2, P3, and P6 but nearly perpendicular to it for particle P4. The magnetization curves can be qualitatively understood and fitted within a simple Stoner–Wohlfarth model^27^ (discussed in more detail in the Supporting Information; see Figures S3–S6), which assumes that size and shape anisotropy determine the energy barrier between two opposite magnetization states. Comparison to more advanced models^28,29^ would require more knowledge about each individual nanoparticle. From this analysis, we fitted the magnetization curves of P2–P4 and P6 (see Figure S7) with aspect ratios (1.2, 1.4, 1.8, and 1.2) and easy axis angles (50°, 50°, 90°, and 40°) with the applied magnetic field, respectively. The magnetization curves of 32 more single particles are presented in Figures S8 and S9 of the Supporting Information. The low slope of particle P4 is assigned to a high aspect ratio of the particle and to the orientation of its long, easy axis nearly in the sample plane. It is important to note that a ferromagnetic particle with its easy axis perpendicular to the applied magnetic field would show a similar magnetization curve (see details in Supporting Information, Figure S22). Particle P1 shows an intermediate behavior, suggesting that magnetic switching might occur during the measurement.

As is apparent from the fluctuations in the MCD signal of particle P1 between positive and negative values (see Figure 1B), it behaves differently from the other particles, which mostly display stable MCD signals. This is confirmed by the large spread of positive and negative *g*_CD_ for particle P1 at small field values (see the image in Figure 1A and magnetization curve in Figure 1E). This behavior is absent for particles P2–P6. The fluctuations of the MCD signal of particle P1 suggest single-particle magnetization switching. We recorded a 100 s MCD time trace of P1 (Figure 2A) without an applied field and indeed found multiple switching events separated by several seconds on average. Time traces of MCD signals of particles P2–P6 do not show any magnetization switching (see Figure S21). We also measured linear dichroism (LD) signals of P1, which do not show any switching (see Figure S10). As the magnetite particles are mostly about 20 nm in size, we assume that our particles have a single magnetic domain, where exchange energy is minimized by alignment of all spins, producing a macrospin (see the calculation of the critical radius for a single-domain magnetite nanoparticle in the Supporting Information). We assign the observed switching events to flips of the macro-spin of particle P1 between two (magnetic-field-dependent) antiparallel states, which we label “up” and “down”. From the Néel–Brown theory, we expect switching to be influenced by an applied magnetic field, as we indeed find in our study of the populations of up and down levels.

**Figure 2:**
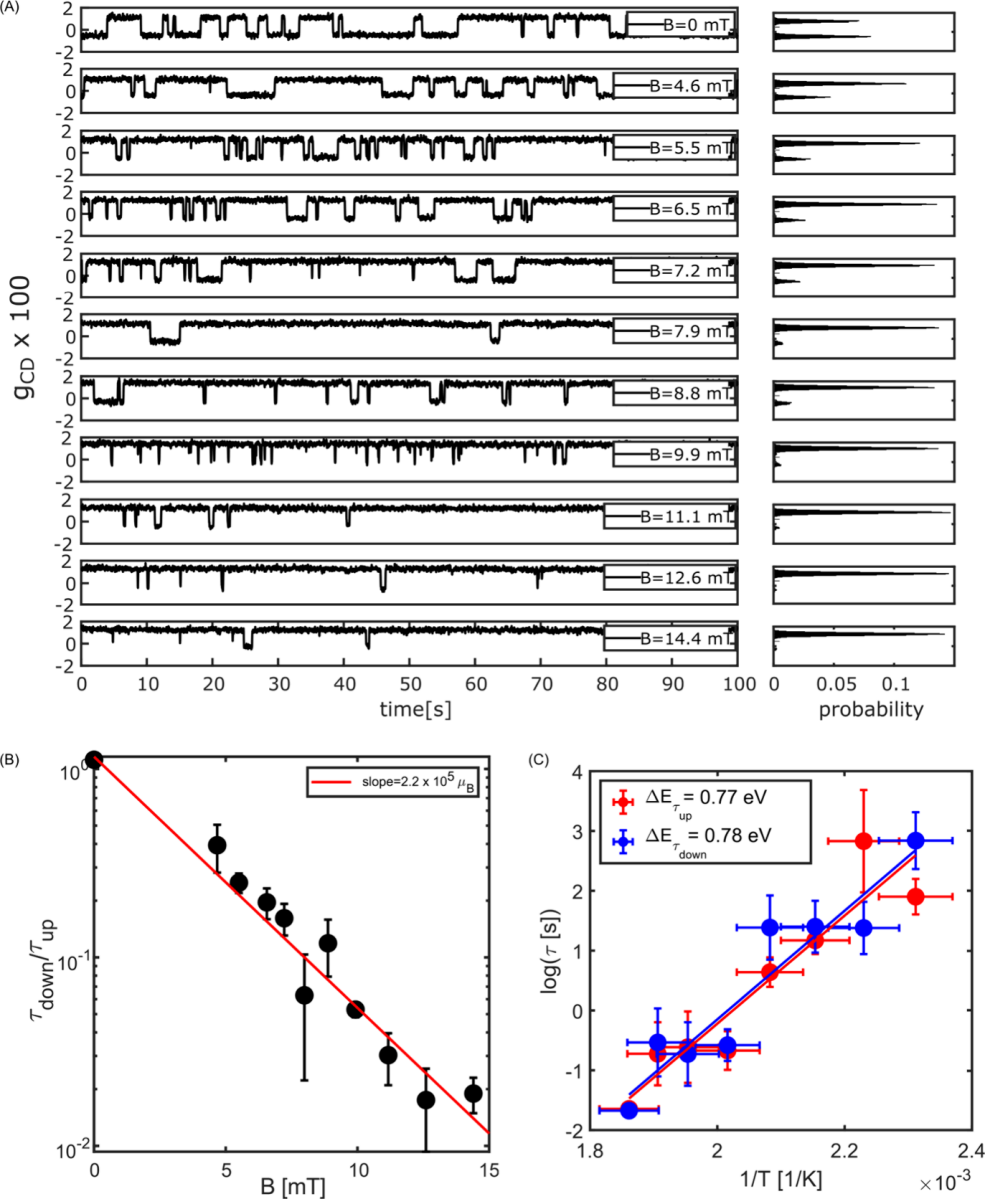
Magnetization switching of a single magnetite nanoparticle (particle P1 of Figure 1) and dependence of the occupation of the two states on magnetic field (*B*) and on temperature (*T*). (A) Magnetization time traces showing switching at different magnetic fields. (B) Ratio of down and up times, τ_down_/τ_up_, versus *B* with a fit according to the Stoner–Wohlfarth model. The fit slope, 2μ cos ψ/*k*_B_T, provides the particle’s magnetic moment. (C) Temperature dependence of up and down times (τ_up_ and τ_down_) fitted with a simple Arrhenius law, which provides an energy barrier of 0.78 ± 0.2 eV mentioned in the inset, and an attempt frequency of about 10^8^ Hz. The *Y* error bars are the standard deviations of three measurements at a given temperature. The *X* error bars are errors in the estimation of temperature considering 5% laser power fluctuations.

Switching time traces of P1 were recorded over 100 s for applied fields varying from 0 to 15 mT and are presented in Figure 2A. The population of the up state (positive *g*_CD_) increases with the magnetic field. Above 15 mT, mostly the up state is occupied, as can also be seen in Figure 1E. A further weak increase in dissymmetry factor *g*_CD_, i.e., in magnetization, takes place at higher fields. We assign it to the gradual orientation of the saturated macro-spin along the external magnetic field. Further details of the Stoner–Wohlfarth fit are given in the Supporting Information (Figure S3). Figure 2A presents histograms of *g*_CD_ for each time trace, which allow us to perform a change-point analysis and to determine the up (τ_up_) and down (τ_down_) residence times (see Figure S11). By fitting the ratio of these times with the Stoner–Wohlfarth model (Figure 2B), we obtain a slope of 2.05 × 10^5^ μ_B_. This slope is approximately given by 2μ cos ψ/*k*_B_*T*, where μ is the magnetic moment, ψ is the angle between the easy axis and the applied magnetic field, *k*_B_ is the Boltzmann constant, and *T* is the absolute temperature. The angle ψ can be determined from a fit to the magnetization curve, as shown in Figure S5, and was found to be about 70 ± 10° in the present case. Therefore, we deduce a magnetic moment of the particle of about 3 × 10^5^ μ_B_, which is reasonably close to the above estimate (4.4 × 10^5^ μ_B_). The slight difference between these two values might arise from a magnetic dead layer close to the nanoparticle’s surface, i.e., a layer of ill-aligned or disordered spins.^30^ A magnetic dead layer with a shell thickness of 1.5 nm would explain the difference between the experimental and expected estimates. The switching behavior of two more particles as a function of applied magnetic field is presented in Figures S12 and S13. Note that we neglect the field produced by other particles nearby because of the large separation between neighbor particles required for the optical resolution of single particles (see an estimation of the interparticle field in the Supporting Information). At a short distance from the permanent magnet, i.e., at high field, the magnetic field may not be very uniform. At distances larger than about 1 cm, however, corresponding to fields of some tens of mT, the field is expected to be very homogeneous over the whole field of view and the Hall probe measurement to be reliable (see Figure S20 and associated discussion).

The Néel–Brown theory^23^ of superparamagnetism assigns macro-spin switching to activated barrier crossing, with a rate following an Arrhenius dependence on temperature. To vary the temperature in our measurements, we varied the laser power of the probe beam, which is tightly focused on the particle under study (the choice of probe versus heating beam was dictated by technical considerations). Based on literature values of the absorption of magnetite and on COMSOL simulations we estimated the temperature of the single particle P1 to vary from 432 to 537 K (with an inaccuracy of about 15 K) in the range of probe powers we used (see Supporting Information, Figures S14 and S15). We used a heating power of 160 mW, and the probe power was varied from 25 to 58 mW. We present the population ratio of up state and down state as an Arrhenius plot (Figure 2C). However, as will be discussed below, the switching rate was found to fluctuate significantly, even at a fixed temperature. We therefore had to average several measurements for each temperature, causing the fairly large error bars on the rates in Figure 2C. Assuming a simple Arrhenius temperature dependence, i.e., ignoring possible dependences of the attempt frequency and of the barrier energy with temperature, we extract an energy barrier of about 0.78 eV for particle P1 from the slope of Figure 2C, which is considerably higher than the thermal energy *k*_B_*T* (0.04–0.05 eV). Such a large barrier combined with the exponential Arrhenius dependence explains why switching rates cover many orders of magnitude of times within a comparatively narrow range of temperatures. As the barrier parameters themselves may depend on temperature, we stress that our estimate of the energy barrier is only qualitative. Finally, we return to the aforementioned fluctuations in the barrier rate. Figure 3A shows a magnetization switching trace of particle D3 (Supporting Information) over a duration long enough to observe hundreds of switching events. The switching behavior is obviously much faster in the time interval between 300 and 600 s than at the beginning and end of the trace, although experimental conditions did not change. In this context, it is important to briefly address the stability of the heating laser’s output. Laser power fluctuations do not exceed 10% in relative value, and their characteristic times are seconds or less. Therefore, power fluctuations cannot explain the large, sudden, and long-lived rate changes displayed in Figure 3. Similar rate changes of an activated process are well-known in single-molecule traces of complex systems such as enzymes under the concept of dynamical heterogeneity.^31^ Histograms of residence times τ_up_ and τ_down_ in the two states are shown in Figure 3B,C. These histograms present a clear excess of events at long times compared to single exponentials, as shown in the insets. As the distribution of switching rates leads to slower-than-exponential decay at long times, we fitted these histograms with stretched exponentials with stretching exponents of 0.6 for τ_up_ and τ_down_. To further prove dynamical heterogeneity, we have used a statistical tool^32^ developed earlier for single protein molecules. After coarse-graining the trace by averaging ten consecutive up times and down times to reduce statistical fluctuations, we correlate consecutive averaged times for up and down states, separately. The corresponding scatter plots are displayed on logarithmic scales in Figure 3D,E. A simulation with an exponential distribution of times gives the correlated points displayed as green dots in Figure 3D,E. The many experimental points falling well outside the green areas confirm that the switching rate itself fluctuates strongly. Similar results are found for another particle monitored over several hours (see Figure S16). This particle started as superparamagnetic and then switched to a ferromagnetic behavior, as we verified by measuring a hysteresis loop. After 2 h without exposure to laser light, the particle returned to its initial superparamagnetic behavior and gradually drifted toward ferromagnetism again. After 2 days in the dark, the particle had returned to a superparamagnetic state.

**Figure 3:**
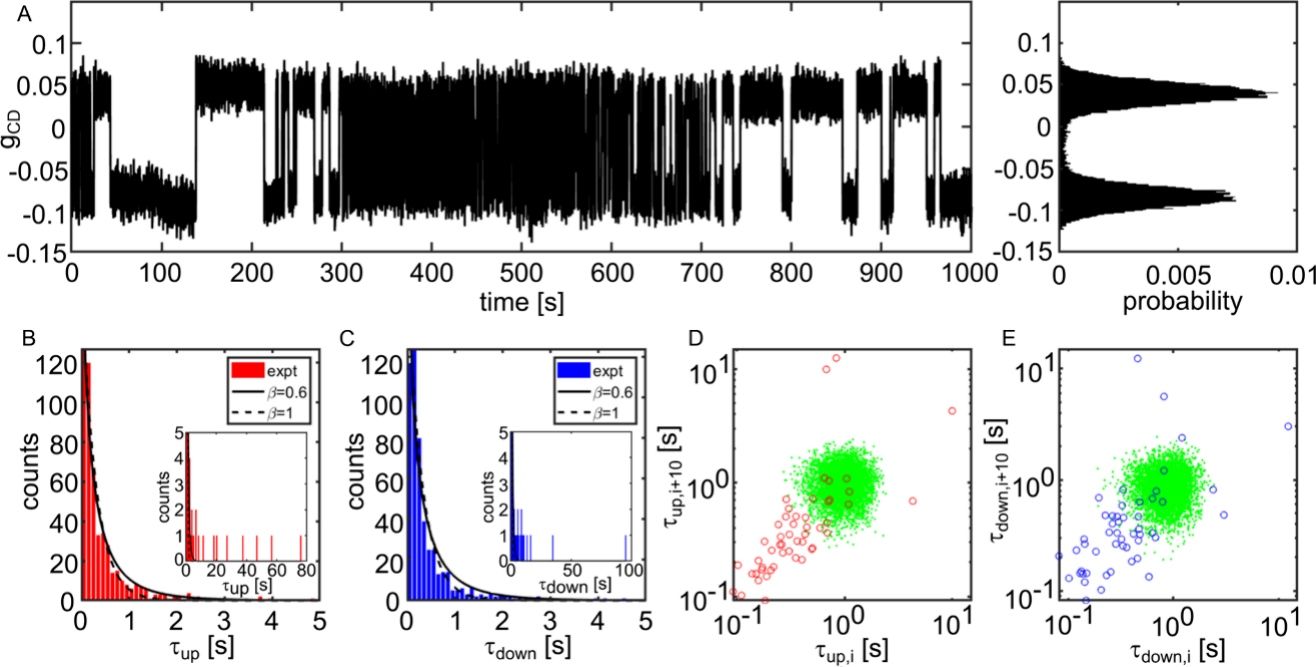
Dynamical heterogeneity of magnetization switching of particle D3 from the Supporting Information (see also a similar plot for particle P1 in the Supporting Information, section 25). (A) Time trace of magnetization switching over 1000 s. The corresponding histogram of *g*_CD_ is shown on the right. (B, C) Histograms of τ_up_ and τ_down_ with stretched-exponential fits (stretching exponents β given in insets). The insets show a few events with long durations that cannot be properly fitted by (stretched) exponential decays. (D, E) Empty circles in correlation plots of successive averages of τ_up_ and τ_down_, averaged over ten successive events. The clouds of green dots are obtained by the simulation of a single-exponential switching process with the same average time. Deviation of the experimental points from the green cloud highlights the strong dynamical heterogeneity of the trace (details in the main text). Note the logarithmic scales of the times.

Dynamical heterogeneity is most often seen as arising from variations of the reaction barrier, through slow conformational changes for proteins,^31,32^ or through variation of the energy landscape in glassy systems,^33^ or through changes of the magnetic energy landscape in the case of magnetic nanoparticles.^9,14,34^ A transition from ferromagnetism to superparamagnetism has been reported previously^34^ for a single iron nanoparticle. However, that study did not report any long time trace with many switching events to support dynamical heterogeneity. In the specific case of magnetite, we speculate that the oxidation state of some iron ions may change through electron transfer or upon oxidation in air, particularly at elevated temperatures (up to 500 K) caused by laser heating. The resulting changes in the spatial distribution of Fe^2+^ and Fe^3+^ ions or in the surface binding of ligands by photo- (or temperature-) driven chemistry could change the magnetic energy landscape.^9,15,35−37^ Additional experiments, such as the removal of organic ligands by plasma etching, or ALD coating the particles with 5 nm of HfO_2_ (see Figures S17–S19) did not clearly indicate any surface origin of the dynamical heterogeneity. Further experiments are needed to explore the role of experimental parameters in switching barrier fluctuations.

In this work, we have imaged and studied individual single-domain magnetite nanoparticles 20 nm in diameter by purely optical means. This is an improvement of about 4 orders of magnitude compared to our previous study of single multidomain magnetite nanoparticulate clusters of 400 nm in diameter. The detection sensitivity reaches about 4 × 10^4^ μ_B_. Although our 20 nm magnetite particles had similar sizes, they turned out to be ferromagnetic or superparamagnetic or to switch between two antiparallel magnetization states on time scales of milliseconds to minutes. Such information has so far been hidden in ensemble-averaged experiments. The various magnetization curves of single nanoparticles were explained within a simple Stoner–Wohlfarth model, with the anisotropy aspect ratio and the angle of the easy magnetization axis as the only fit parameters, adjusted for each particle. The magnetic-field dependence of thermally assisted switching provided us with an estimated magnetic moment of a single magnetite nanoparticle of 10^5^ μ_B_. An anisotropy energy barrier of about 0.8 eV was obtained from the temperature dependence of the switching. The switching rate was found to fluctuate over time, revealing dynamic heterogeneity found earlier in other complex nanometer-scale systems. Such a dynamical heterogeneity commonly observed in protein dynamics or in glassy systems is new and surprising for purely mineral nanoparticles. Our experiments thus demonstrate the versatility of our technique and the rich information that can be gained at the single-particle level by optical means alone. They open new possibilities to explore the influence of composition, surfaces, and defects on nanomagnetic switching, or to study new devices for thermomagnetic actuation, such as antiferromagnetic nanoplatelets.^38^

## Supporting Information Available

Photothermal imaging of single magnetite nanoparticles in hexadecane, TEM images of single magnetite nanoparticles, Stoner–Wohlfarth model, magnetization curves of particles P2–P4 and P6 with Stoner–Wohlfarth fits, magnetization curves of 32 single magnetite particles, time traces of PT, CD, LD, and LD45 signals for particle P1, threshold analysis of field-dependent switching events for particle P1, field-dependent time traces of particles P7 and P8, temperature estimation, temperature dependence of the switching events of particle P1, dynamical heterogeneity of a particle measured over several hours, dynamical heterogeneity of UV-plasma-cleaned particles, dynamical heterogeneity of a particle covered with HfO_2_, dynamical heterogeneity of a particle which was plasma-cleaned and covered with HfO_2_, discussion of the terminology of the *g*-factor and the dissymmetry factor *g*_CD_, estimation of interparticle magnetic field in our sample, magnetic field vs distance, basic principle of the PT MCD technique, detection sensitivity, possible applications of PT MCD, calculation of the critical radius for a single-domain magnetite particle, time traces of MCD signals for particles P1–P6 as labeled in Figure 1, attempt frequency obtained from the Arrhenius fit of temperature-dependent switching of particle P1 in the main text, magnetization curve of a ferromagnetic particle, analysis of dynamical heterogeneity of another time trace similar to that shown in Figure 3, size distribution of single magnetite nanoparticles, absorption spectra of magnetite, sample preparation, and optical setup (PDF)
