# Supplementary material for: Magnetization Switching of Single Magnetite Nanoparticles Monitored Optically
Source: Nano Lett. 2024 Jul 30;24(32):9861–7. doi: 10.1021/acs.nanolett.4c01850 (PMC11328164; doi:10.1021/acs.nanolett.4c01850)
Supplement: Supplementary file 1 — nl4c01850_si_001.pdf [file nl4c01850_si_001.pdf]

Supplementary Material for

Magnetization Switching of Single Magnetite Nanoparticles Monitored Optically

Subhasis Adhikari[1]†*, Yonghui Wang[1,2]†, Patrick Spaeth[1], Francesca Scalerandi[3], Wiebke Albrecht[3], Junyan Liu[2], Michel Orrit[1]*

† These authors contributed equally to this work

[1] Huygens-Kamerlingh Onnes Laboratory, Leiden University; 2300 RA Leiden, The Netherlands

[2] School of Mechatronics Engineering, Harbin Institute of Technology; Harbin 150001, P. R. China

[3] Department of Sustainable Energy Materials, AMOLF; Science Park 104, 1098 XG Amsterdam, The Netherlands

*Corresponding author. Email: orrit@physics.leidenuniv.nl, Adhikari@physics.leidenuniv.nl

1. Photothermal imaging of single magnetite nanoparticles in hexadecane

To estimate the size of a single magnetite nanoparticle from photothermal measurements, we performed photothermal imaging of many single magnetite nanoparticles as shown in Figure S1. A total of 465 single magnetite nanoparticles were detected and a histogram of cubic root of their photothermal signals is shown in the same figure. Aggregates are distinguished by their very strong photothermal signals (see uncircled spots in Fig. S1) and were not considered in the histogram. The photothermal signal is proportional to a particle's volume and thus, the cubic root of the photothermal signal is proportional to the average diameter of the particle. The histogram of the cubic root of photothermal signals corresponds to the size distribution obtained from the TEM measurements. The peak value of the photothermal histogram corresponds to the peak of value of the size histogram i.e., about 19 nm as obtained from TEM measurements shown in Figure S2. A histogram of signal-to-background ratios of 465 single magnetite nanoparticles is also shown in Figure S1. The peak value is about 40 for 19-nm-diameter particles. Such a high signal-to-background ratio indicates that magnetite nanoparticles smaller than 19 nm can be detected in photothermal imaging.

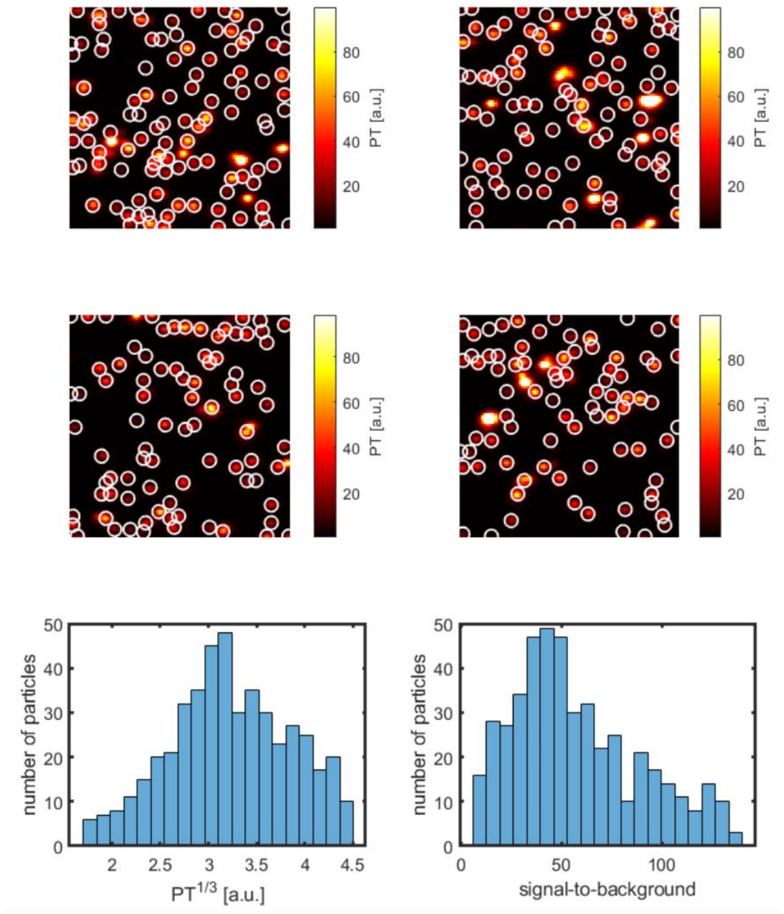

Figure S1: Photothermal measurements of single magnetite particles in four different regions of the sample. Single particles and aggregates are distinguished by their PT strength. Single particles are marked with white circles. The distribution of the cubic root of maximum photothermal signals of 465 single particles is shown. The peak value of about 3 a. u. corresponds to magnetite particles about 19 nm as shown in Fig. S2. The size mentioned in Figure 1 in the main text is calculated based on this calibration measurement. The cubic root of the PT signal corresponds to the average size of a particle. A histogram of signal-to-background ratios for the 465 single particles is also shown.

2. TEM images of single magnetite nanoparticles

To get an idea of the size and shape of single magnetite nanoparticles and of their crystallinity, we performed transmission electron microscopy (TEM) imaging as shown in Figure S2. From the TEM images, it is evident that most particles are single-crystalline and heterogeneous in their sizes and shapes. Most particles have asymmetric shapes. To calculate the size of each particle, it is assimilated to a prolate ellipsoid and its volume is calculated. From the ellipsoid volume, the effective average diameter of the particle is calculated as that of the sphere with the same volume. The average diameter obtained from 38 single particles is 19 nm ± 0.5 nm, i.e., close to the size of about 20 nm provided by the manufacturer.

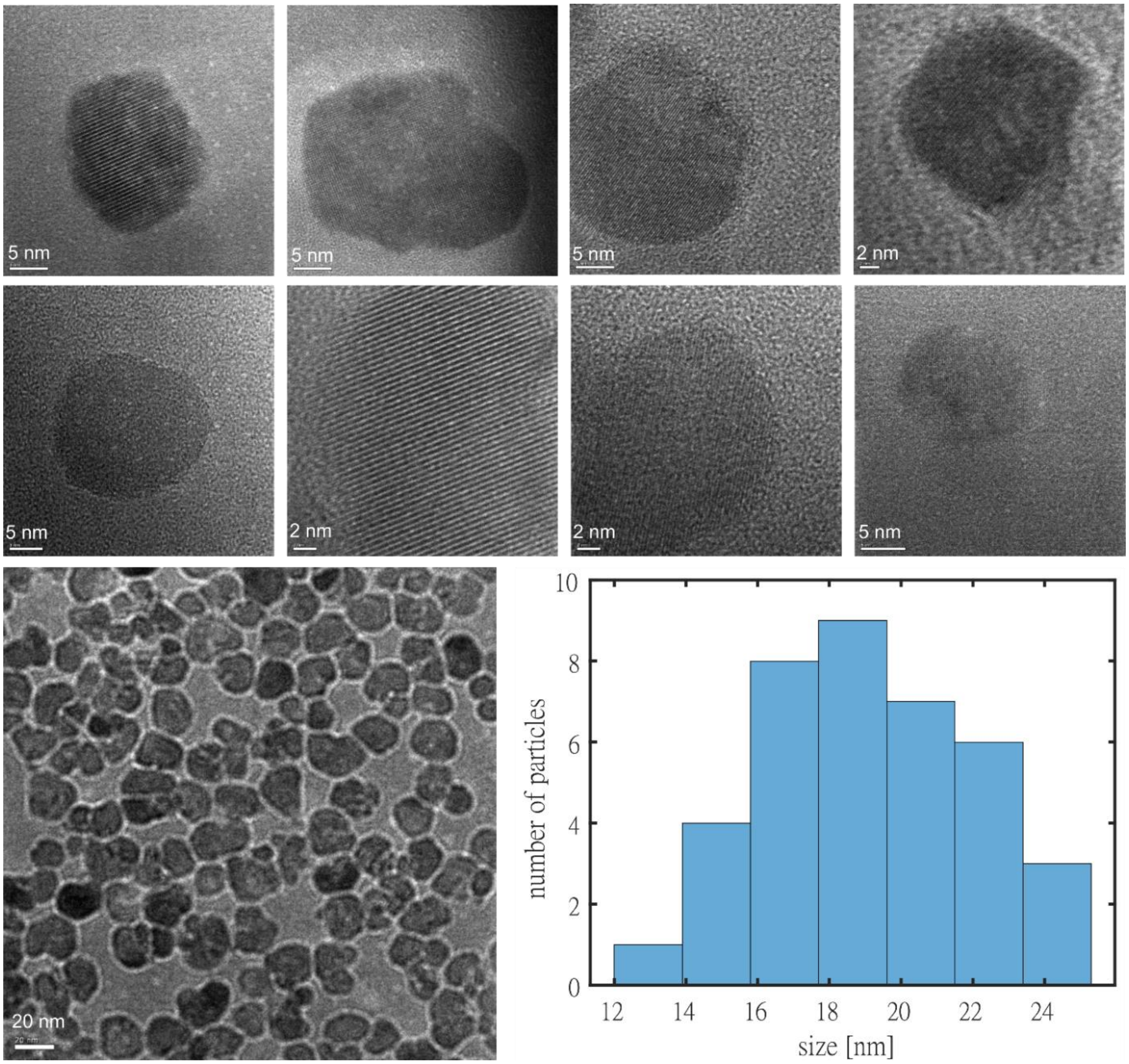

Figure S2: The top two rows show TEM images of eight single-crystalline magnetite nanoparticles. (Bottom left) TEM image of many single magnetite nanoparticles showing heterogeneity in size and shape. The image was taken slightly out-of-focus for easier identification of particle boundaries. (Bottom right) Histogram of effective diameters of single particles determined from the TEM data.

3. Stoner-Wohlfarth model

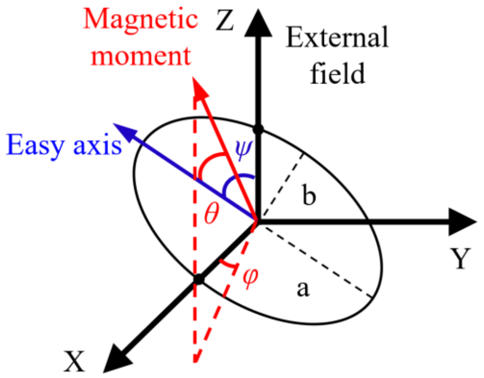

Figure S3: Schematic representation of a prolate ellipsoid with the angles used for the Stoner-Wohlfarth model. θ is the angle between the easy axis and the magnetic moment, ψ is the angle between the external magnetic field and the easy axis and φ is the azimuthal angle. a and b are the lengths of the semi-major and semi-minor axes. The aspect ratio is a/b.

Considering a magnetite nanoparticle as a prolate spheroid (Figure S3), the energy of a magnetic state can be calculated using the Stoner-Wohlfarth model as:

$$E = K_{eff}V\sin^2\theta - \mu B(\cos\theta\cos\psi + \sin\theta\sin\psi\cos\varphi),$$

where V is the volume of the particle, θ is the angle between the easy axis and the magnetic moment and φ is the azimuth angle of the magnetic moment μ ($\mu = M_sV$, where M_s is the saturation magnetization), B is the applied external field, ψ is the angle between the easy axis and the external magnetic field. The effective anisotropy constant K_{eff} can be written as $K_{eff} = K_1 + \frac{\mu_0 M_s^2(N_x - N_z)}{2}$, where μ_0 is the vacuum permeability, and K_1 possibly accounts for anisotropy contributions not arising from the shape (such as the magneto-crystalline and surface anisotropy), which we neglect hereafter ($K_1=0$). The second part is the shape anisotropy which we assume to dominate in our case. N_x and N_z are the demagnetization factors of the nanoparticle. For spheroidal nanoparticles, the shape anisotropy is determined by the aspect ratio $k = a/b$, yielding the following demagnetization factors for the cases of oblate, sphere, and prolate spheroids[1]:

$$N_z = \frac{1}{1-k^2}\left[1 - \frac{k}{\sqrt{1-k^2}}\arccos(k)\right], k < 1$$

$$N_z = \frac{1}{3}, \quad k = 1$$

$$N_z = \frac{1}{k^2-1}\left[\frac{k}{\sqrt{k^2-1}}\text{arccosh}(k) - 1\right], k > 1$$

$$N_x = (1 - N_z)/2$$

Hereafter, we model our nanoparticles as prolate spheroids, as the case of oblate spheroids presenting two degenerate axes is marginal. Under thermal equilibrium conditions ($\tau < t_m$ where τ is the residence time in a certain magnetic state and t_m is the measurement time), the average magnetization with a given orientation (ψ) of the easy axis with respect to the external magnetic field direction is given by:[2]

$$m(\psi) = \frac{M}{M_S} = < \cos(\theta - \psi) > = \frac{\iint (\cos(\theta\text{-}\psi)\cdot \exp\left(-\frac{E}{k_B T}\right)\sin\theta \mathrm{d}\theta \mathrm{d}\varphi}{\iint \exp\left(-\frac{E}{k_B T}\right)\sin\theta \mathrm{d}\theta \mathrm{d}\varphi},$$

where M_s is the saturation magnetization, k_B is the Boltzmann constant and T is the absolute temperature. We can get average magnetization curves for different aspect ratios and easy axis orientations using the above equation. Figure 1 of the main text shows different cases of magnetization curves. The case of particle P6 shows a quick saturation of the magnetization for a weak field, with nearly no further increase when the field is further raised. We assign this curve to a particle with its easy axis nearly collinear with the applied field, leading to an early saturation. Particles P2, P3, and to a lesser extent P1, show a steep initial magnetization increase for a weak field, followed by a more gradual increase for higher fields. We assign this behavior to particles with tilted easy axes, which first saturate their magnetic moment along the easy axis, then rotate their magnetization axis upon competition of the anisotropy with the Zeeman energy. In that case, a first saturation along the easy axis gives a component $M_s \cos\psi$ along the applied field, whereas a further increase of the field moves the magnetization towards the applied field, ending up with projection M_s on the applied field. Finally, the case of P4 is assigned to a particle whose easy axis is nearly perpendicular to the applied field. No first saturation can be distinguished, and the magnetization gradually rotates from horizontal to vertical along the applied field. For aspect ratios close to 1, the nearly spherical particle magnetizes along the applied field, producing a magnetization curve very similar to that of P6. These behaviors are exactly obtained in the Stoner-Wohlfarth simulations presented in Figure S4, so that angle and aspect ratio can often be determined by visual comparison of the measured magnetization curves to the simulations.

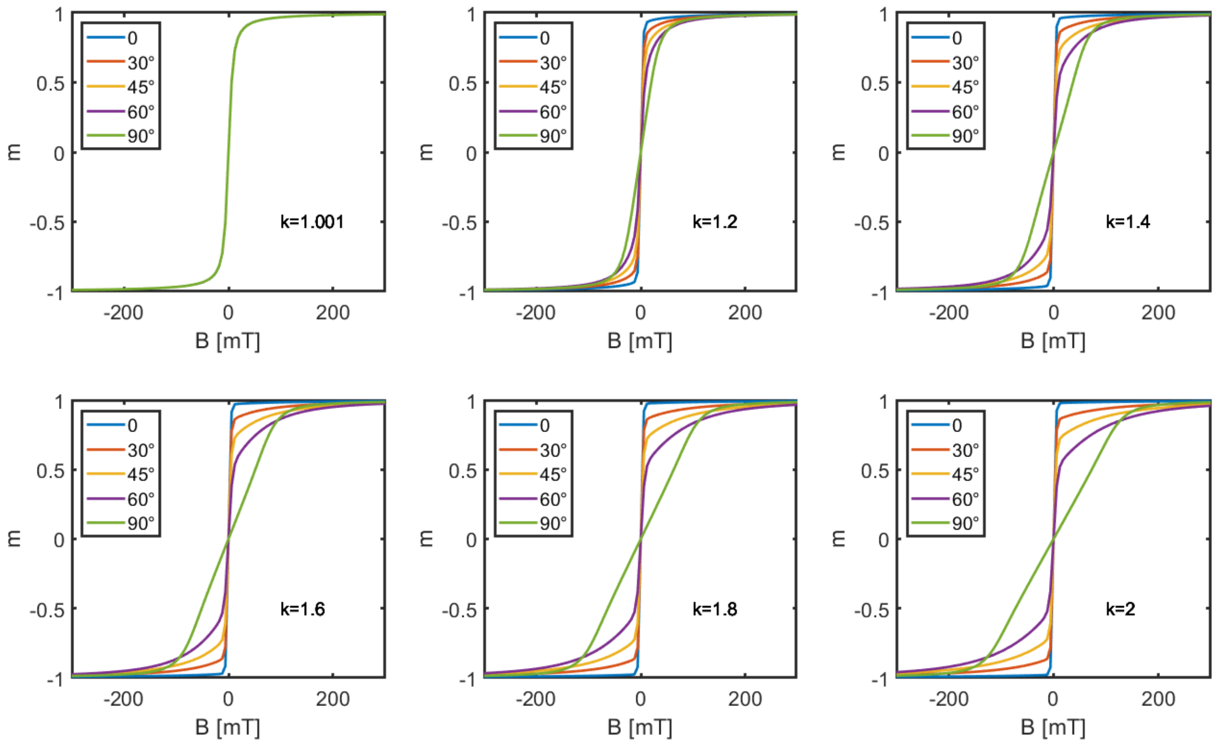

Figure S4: Magnetization curves simulated using the Stoner-Wohlfarth model for particles with different aspect ratios (k) and angles (ψ) of the easy axis with respect to the external magnetic field.

If the temperature is too low to establish thermal equilibrium on time scales much shorter than the measurement, i.e., if the thermal energy $k_B T$ allows occasional crossing only of the energy barrier during the measurement, the magnetization will be seen to switch between two states. Then, the population ratio of the two states i.e., the ratio of residence times in the two states follows from Boltzmann statistics as:

$$\frac{\tau_1}{\tau_2} = \exp\left(\frac{E_2 - E_1}{k_B T}\right).$$

Now, using the energy from the Stoner-Wohlfarth model, we can write this ratio for a small enough applied field (i.e., assuming that the applied field is too weak to change the magnetic moment's direction) as:

$$\log\left(\frac{\tau_1}{\tau_2}\right) = \frac{2\mu\cos\psi}{k_B T} B.$$

It turns out that the plots of $\log\left(\frac{\tau_1}{\tau_2}\right)$ vs. B are very close to straight lines in all cases. Therefore, if we plot $\log\left(\frac{\tau_1}{\tau_2}\right)$ vs. B (Figure S5), we can calculate the magnetic moment (μ_{fit}) as

$$\mu_{\text{fit}} = \frac{k_B T}{2\cos\psi} \frac{d}{dB}\left(\log\left(\frac{\tau_1}{\tau_2}\right)\right)$$

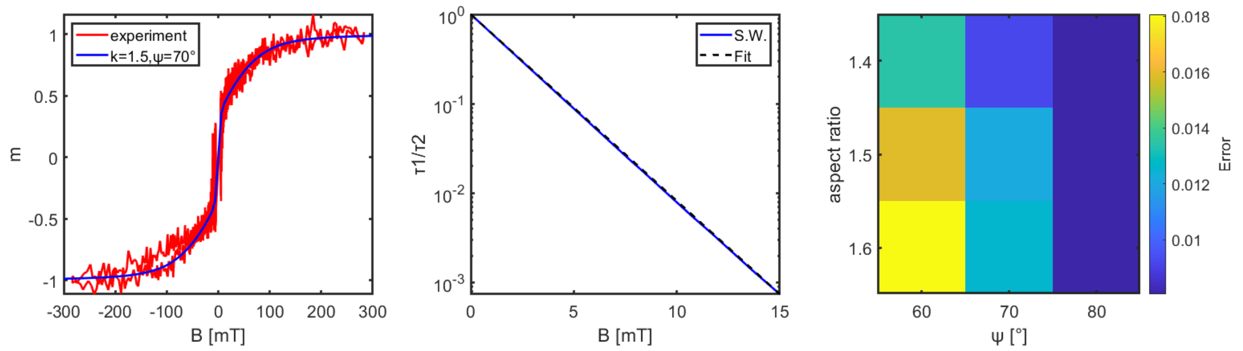

Figure S5: (Left) a magnetization curve fitted with the Stoner-Wohlfarth model. The aspect ratio (k) and the angle of the easy axis (ψ) are mentioned in the inset. (Middle) The plot $\log\left(\frac{\tau_1}{\tau_2}\right)$ *vs. the external magnetic field (B) with a linear fit and with the fit using the Stoner-Wohlfarth model. (Right) The error map for the linear fit and the Stoner-Wohlfarth fit for different values of k and ψ.*

Figure S6 shows the energy landscape in the XZ plane under an external magnetic field. When there is zero or weak magnetic field, the magnetic moment prefers aligning to the minimum energy direction i.e. along the easy axis. There are two minimum energy points corresponding to up and down spin states as schematically shown in Figure S6. With the increase in magnetic field strength, the energy diagram shifts towards one minimum and the magnetic moment gradually aligns to the magnetic field direction. The rate of the change in the energy diagram with increase in magnetic field depends on the orientation of the magnetic field with respect to the easy axis, i.e., on angle ψ.

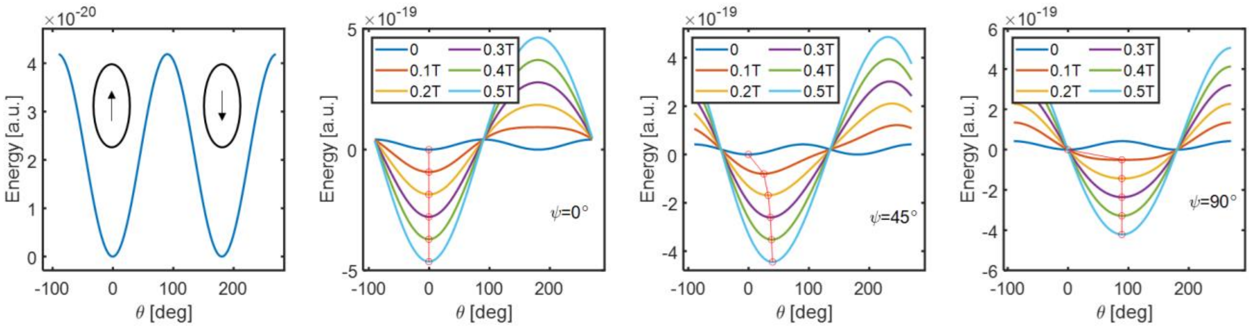

Figure S6: the energy landscape in XZ plane of a single magnetite nanoparticle. (Left) the energy landscape when there is no external magnetic field. (Middle and right) The energy diagram at different field strengths at three different angles of the field orientation w.r.t the easy axis. The red lines with circle show the minimum energy points.

4. Magnetization curves of particles P2, P3, P4 and P6 with Stoner-Wohlfarth fits

Figure S7 shows magnetization curves of particles P2, P3, P4 and P6 as labelled in Figure 1 in the main text. The magnetization curves are fitted with the Stoner-Wohlfarth model considering different aspect ratios (k) and angles between the applied magnetic field and the easy axis (ψ). The optimized values are obtained by minimizing the error of the fit to the experimental data and shown in the insets of center panels in Figure S7.

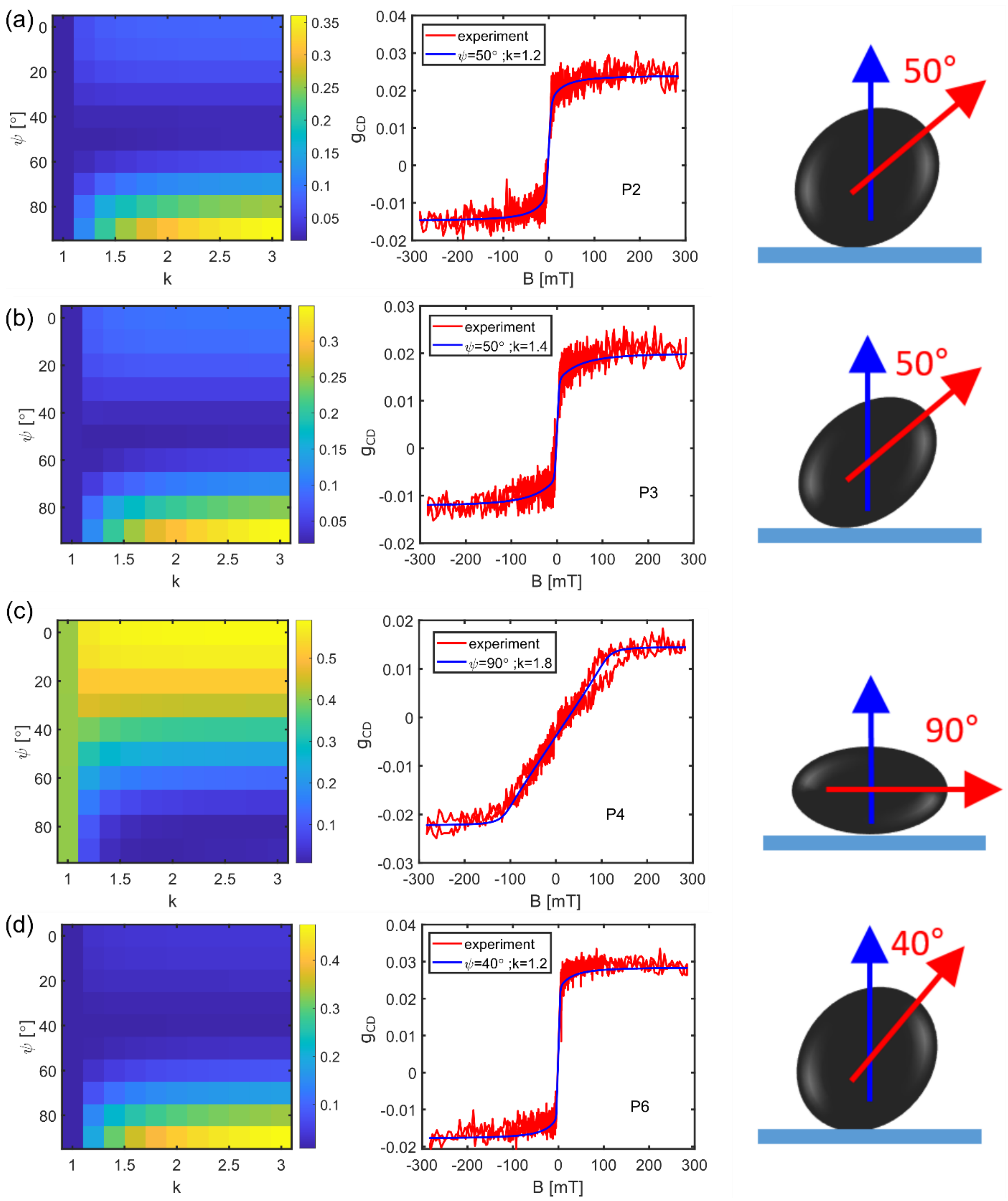

Figure S7: Magnetization curves of four particles (P2, P3, P4 and P6 as labelled in Figure 1 in the main text) with Stoner-Wohlfarth fits. (Left) An error map of the fit from the experimental data at different aspect ratios (k) and angles between the easy axis and the applied field direction (ψ). (Center) The obtained k and ψ values obtained for the minimum error are shown

in the insets and the corresponding Stoner-Wohlfarth fit (blue) is plotted along with the experimental data (red). Particle labels are mentioned. (Right) Schematic representation of the magnetic moment (red) along the prolate particle axis for low enough applied magnetic field (blue).

5. Magnetization curves of 32 single magnetite particles

We measured the magnetization curves of 32 single magnetite nanoparticles as shown in Figure S8 and Figure S9. We categorized them in three types, Type I, Type II and Type III. Type I particles are superparamagnetic i.e., magnetization switching is much faster than our measurement timescale, Type II particles present intermediate behaviors, i.e., magnetization switching within the measurement time and Type III particles are ferro(i)magnetic where magnetization switching is much slower than the experimental timescale. Magnetite is ferrimagnetic, but we will use the usual terminology of superparamagnetism and refer to its ferrimagnetic state as 'ferromagnetic' in the following. The particles that have magnetization curve similar to particle P4 shown in Fig. S7, have in-plane easy axis. It is difficult to distinguish whether these particles are superparamagnetic or ferromagnetic as ferromagnetic particles with in-plane easy axis would also show similar magnetization curves (see later in Fig. S22). These kinds of particles in Fig. S8 and Fig. S9 are termed Type I or Type III.

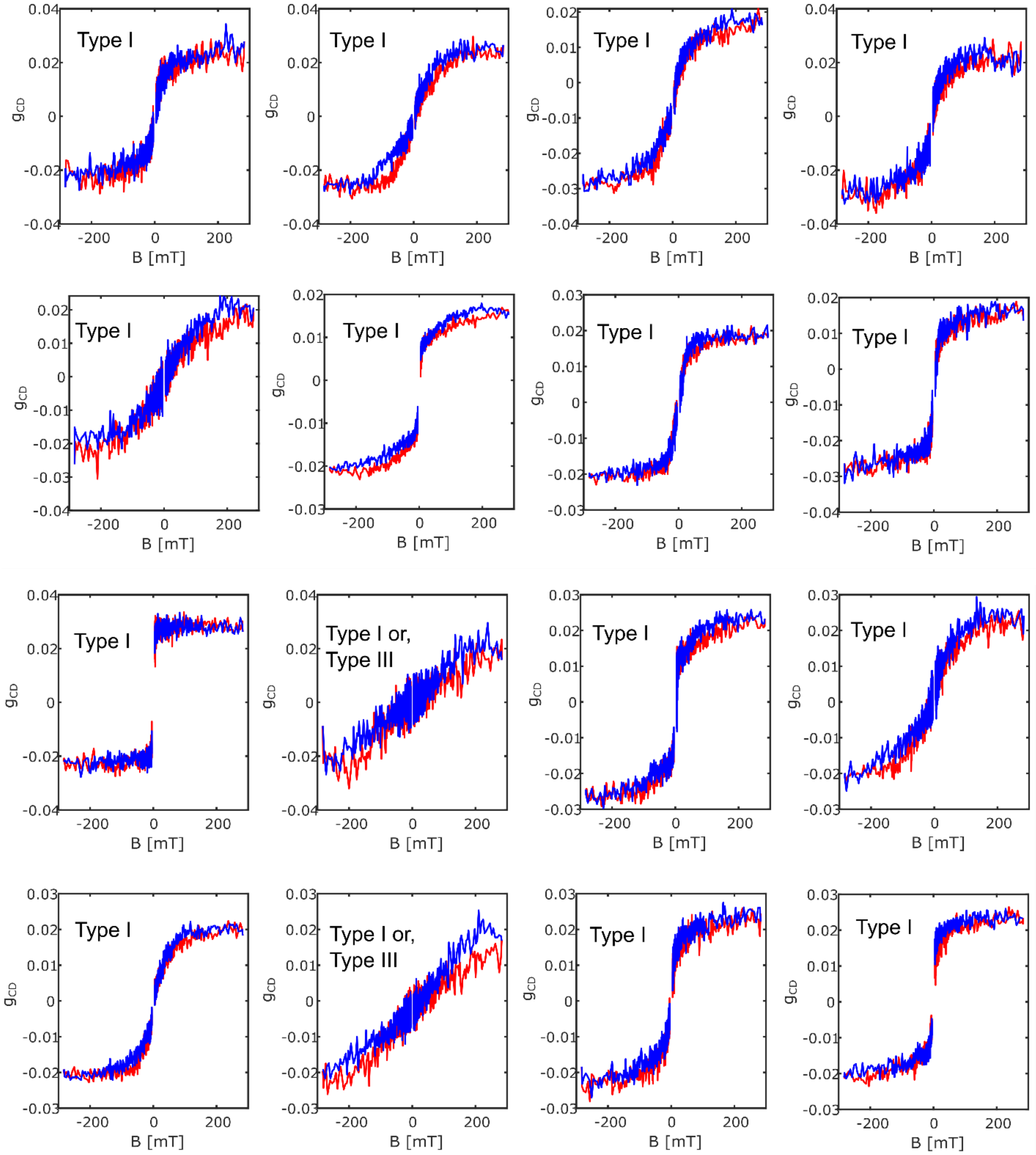

Figure S8: Magnetization curves of 16 single magnetite nanoparticles of Type I and Type III. Red: scan from positive to negative applied magnetic field, blue: scan from negative to positive applied magnetic field.

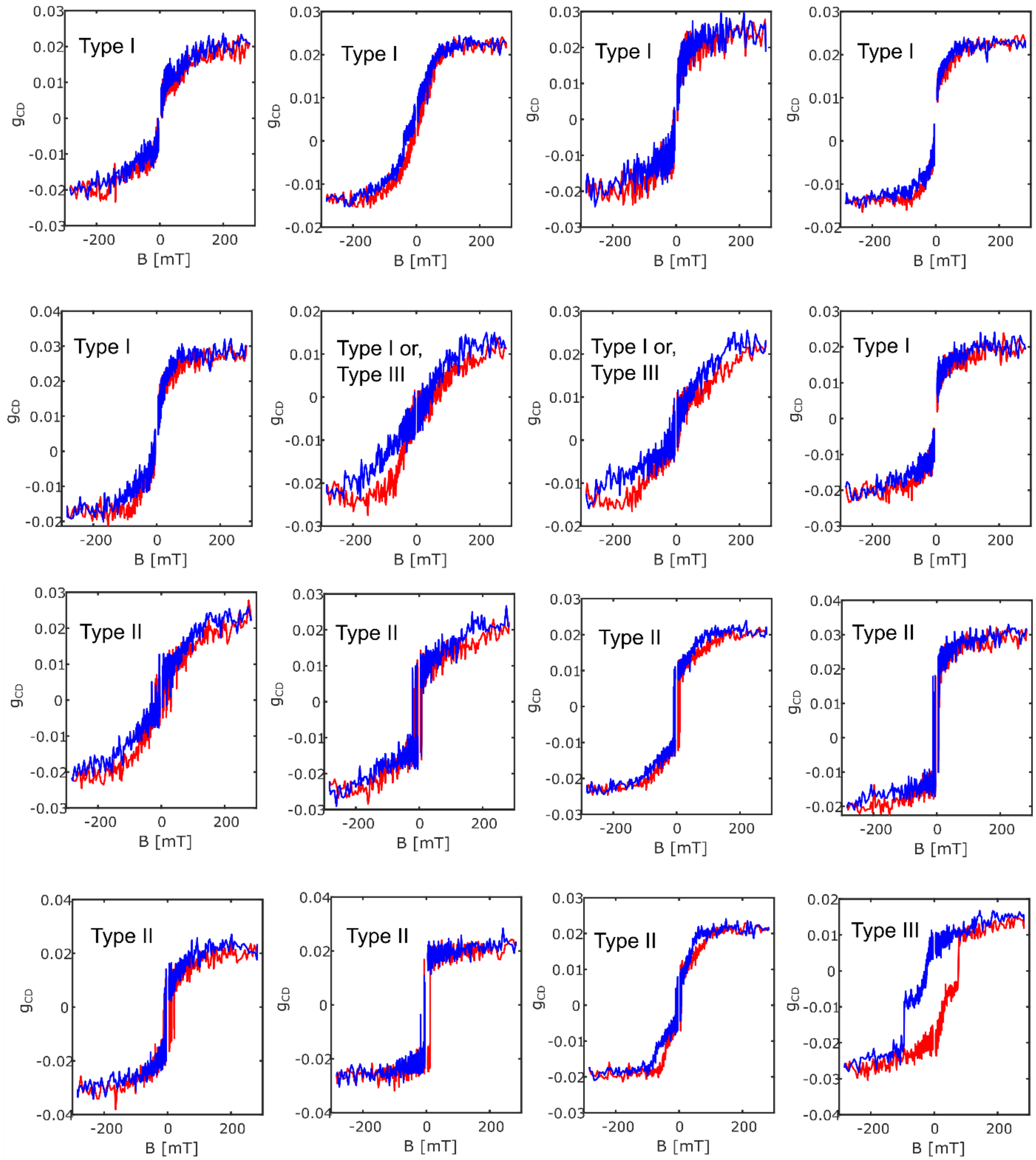

Figure S9: Magnetization curves of 16 single magnetite nanoparticles of three different types, Type I, Type II and Type III. Red: scan from positive to negative applied magnetic field, blue: scan from negative to positive applied magnetic field.

6. Time traces of PT, CD, LD and LD45 signals for particle P1

Figure S10 shows time traces of photothermal absorption (PT), circular dichroism (CD), and linear dichroism at two different polarization orientations, at 0°/90° (LD) and at 45°/-45° (LD45). Although there are some signal fluctuations for the case of PT, LD and LD45, none of them show signal fluctuations between two states. The only trace showing clear switching between two states is the CD time trace.

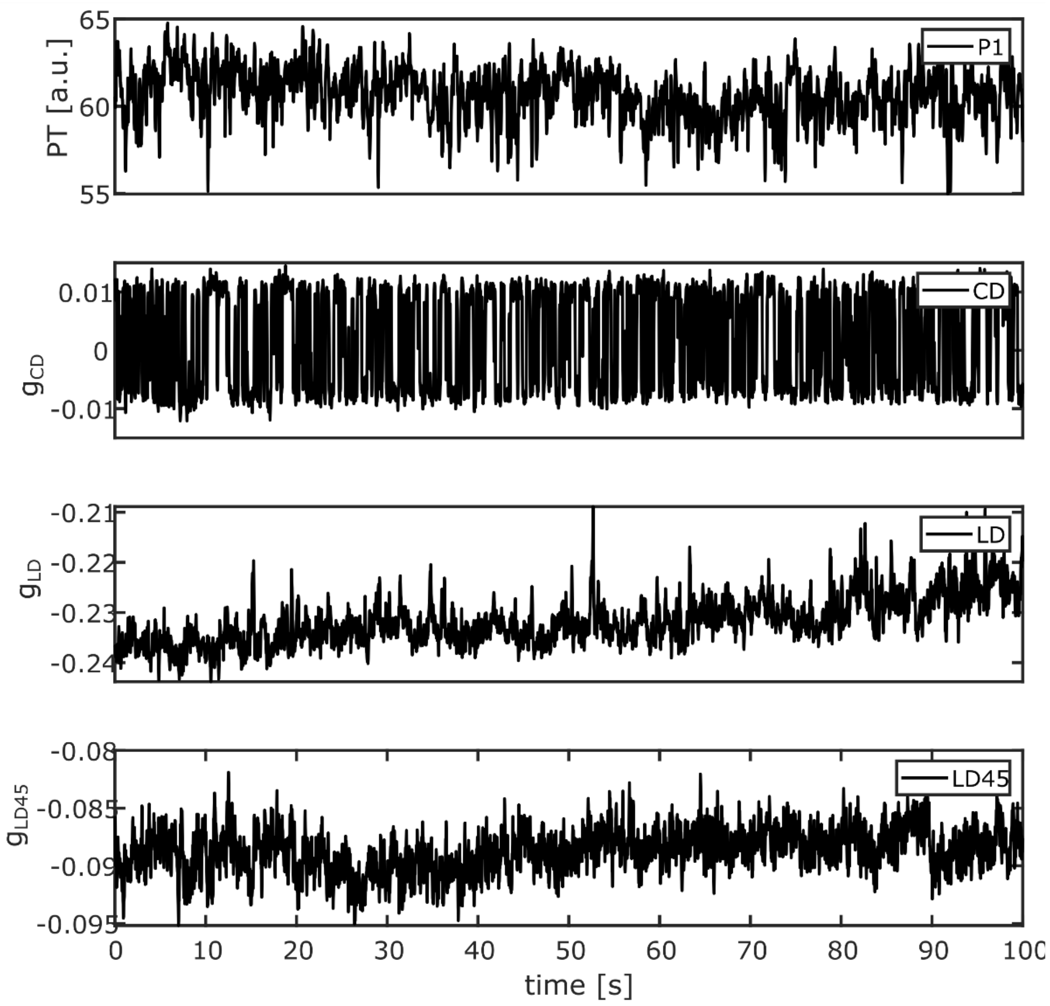

Figure S10: Time traces of PT, CD, LD and LD45 of particle P1 over 100 s. The slight decrease in LD signal at the end of the time trace is due to slight defocusing. We see signal fluctuation between positive and negative values only for the CD time trace.

7. Threshold analysis of field-dependent switching events for particle P1

Magnetization time traces of particle P1 at different magnetic fields presented in Fig.2 are reproduced here with a change-point analysis using the mid-point between the two histogram peaks as threshold (Figure S11). The threshold defines the two different states to calculate the residence times in the two states.

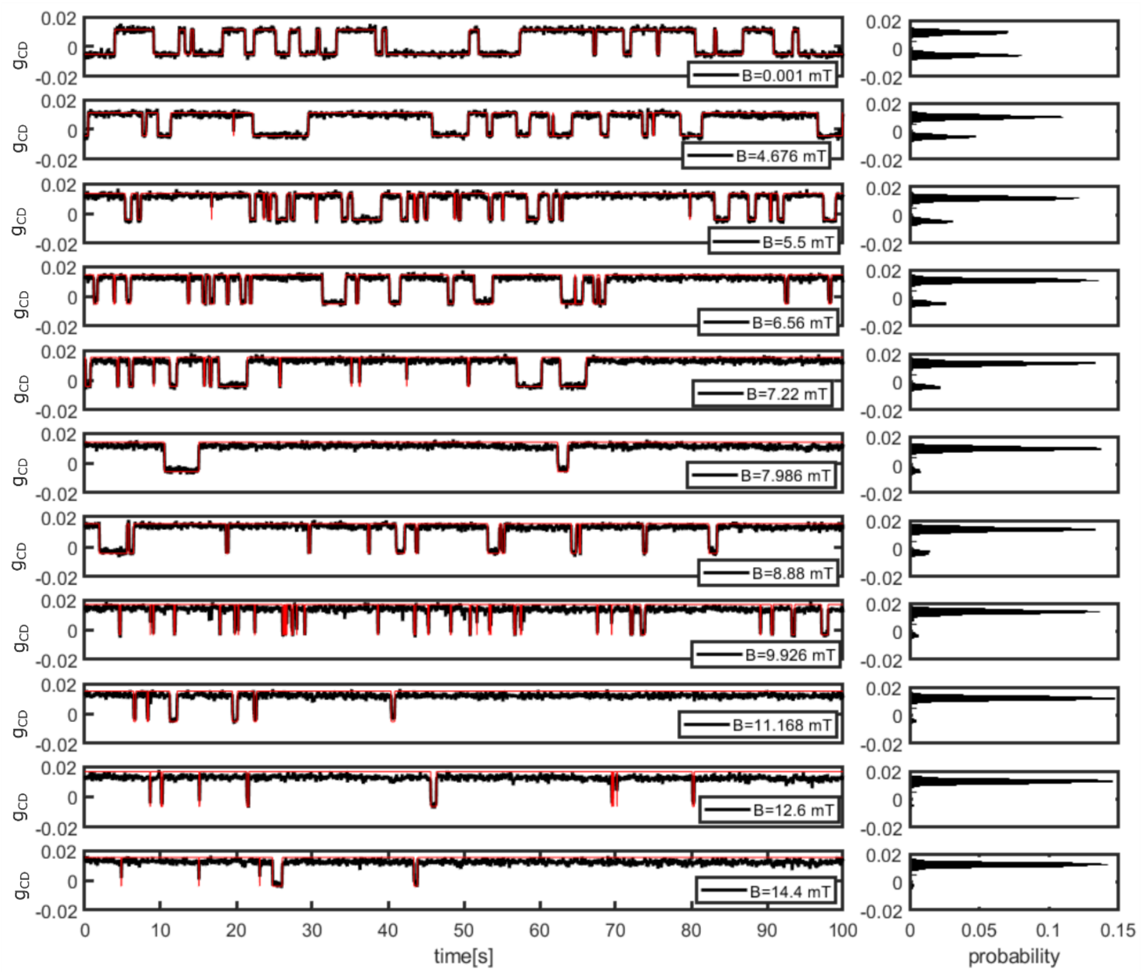

Figure S11: External magnetic-field-dependent time traces of Fig. 2 of the main text. The threshold is defined to distinguish two states. The fits based on this threshold analysis are shown with solid red lines. The histogram of each time trace, providing the occupation probability of each state, is shown on the right. The dwell times in each state are deduced from the change-point analysis.

8. Field-dependent time traces of particles P7 and P8

In the main text, we have shown field-dependent time traces of particle P1 only. Here, we show similar time traces for two more particles, P7 and P8 in Figure S12.

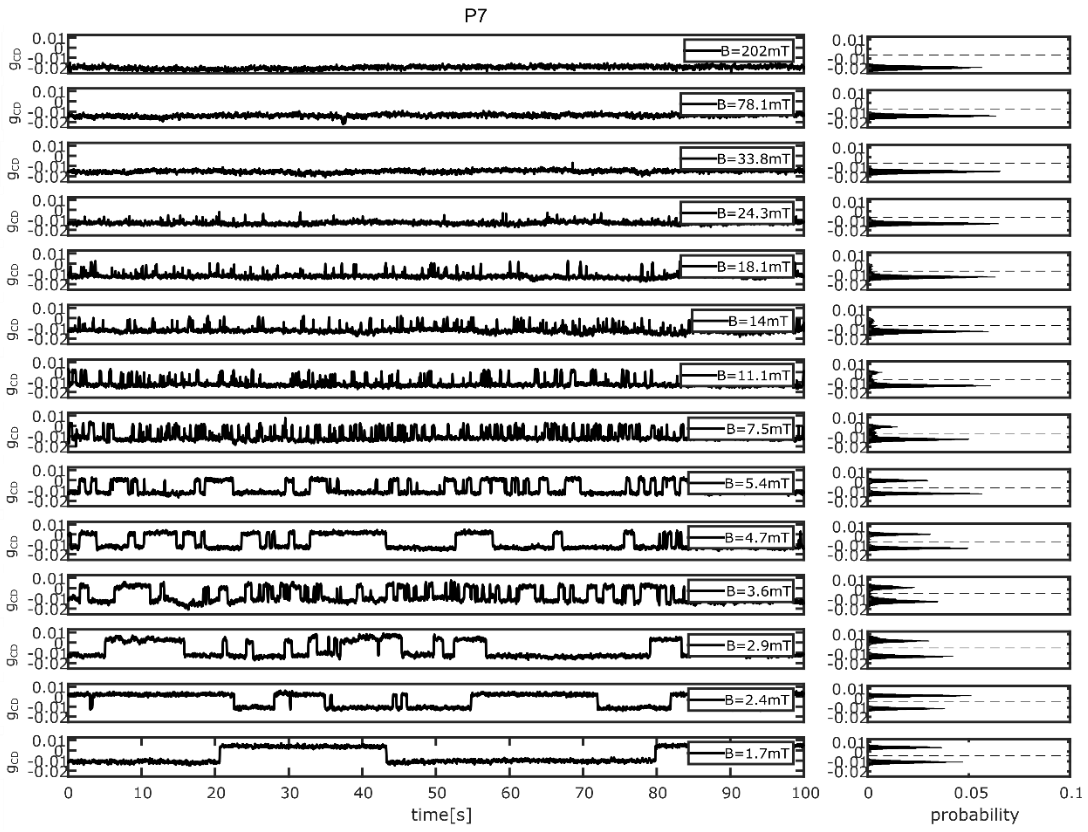

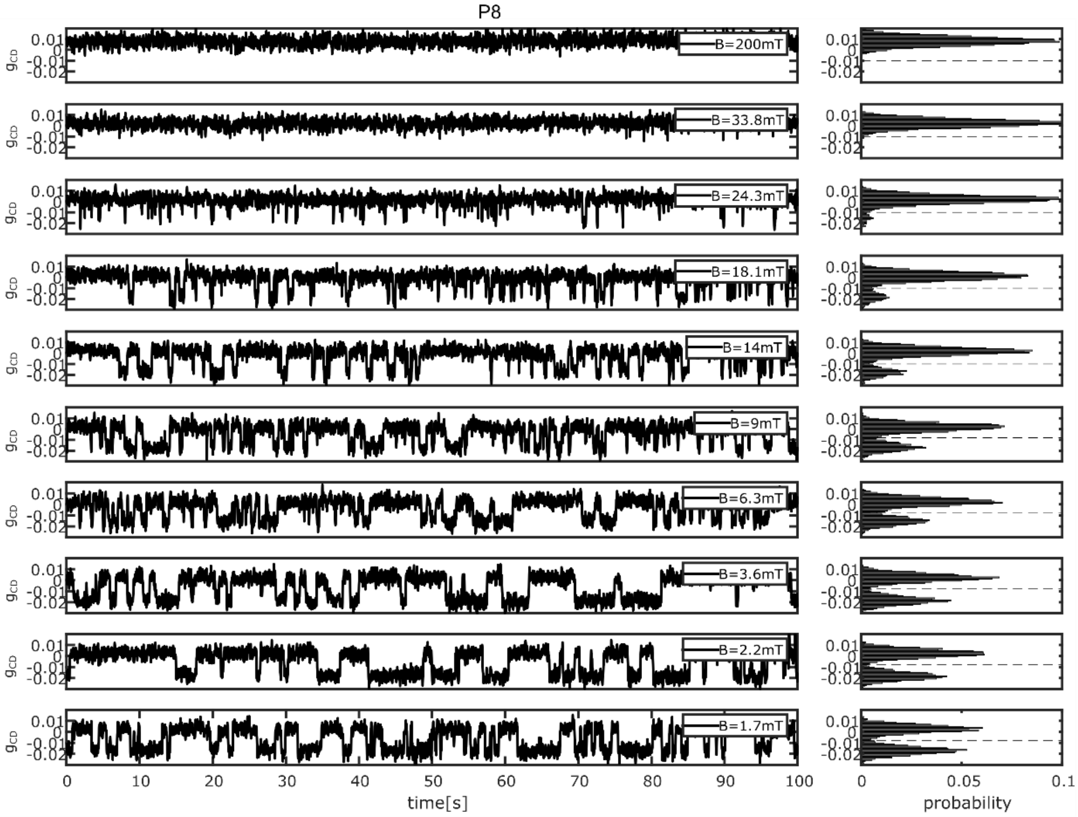

Figure S12: Magnetic-field-dependent time traces of particles P7 and P8.

The ratio of residence times in the two opposite magnetization states of particles P7 and P8 is presented in Figure S13 as a function of the applied magnetic field. For all values of the angle ψ, the Stoner-Wohlfarth model gives a very close-to-linear variation with a slope of $2\mu\cos\psi/k_B T$. Unfortunately, we did not measure the magnetization curves of these two particles and therefore we do not know the value of ψ. As can be seen from the time traces of these two particles, the field required to dominantly populate only one state has a value close to the one found for particle P1 in the main text. Therefore, we assumed ψ to be close to 70° for particles P7 and P8. Considering ψ=70°, the magnetic moments for the P7 and P8 are about 3×10^5 Bohr magnetons and 0.95×10^5 Bohr magnetons. The magnetic moments of P7 and P1 are quite similar, in good agreement with their estimated sizes (23.4 nm diameter for P7 and 24.7 nm for P1). The magnetic moment of particle P8 is about 3 times lower than that of particle P7 which is close to the expected volume ratio, about 4.7. The slight mismatch could be explained by the thickness of their dead layers. As the surface-to-volume ratio is higher for smaller particles, the similar dead-layer thickness would lead to a reduced magnetic moment for smaller particles. The mismatch could also be due to different ψ values as we do not know the exact ψ values for these two particles.

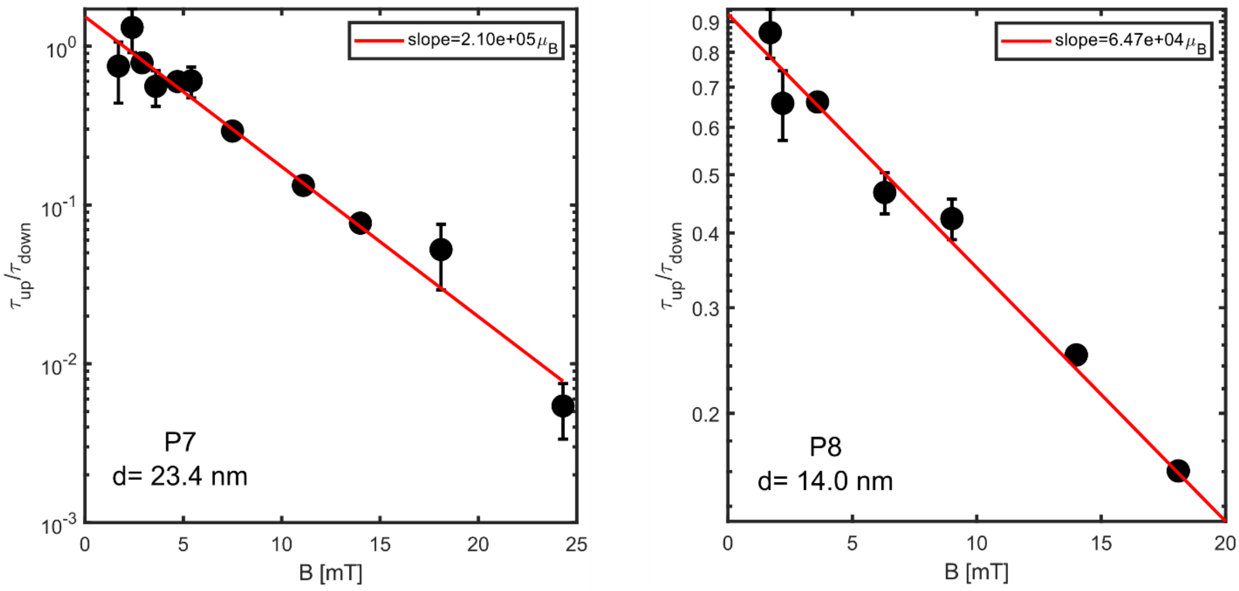

Figure S13: External-field-dependent Boltzmann behavior fitted with the Stoner-Wohlfarth model for particles P7 and P8. The slopes extracted from the fits are 2.1×10^5 and 6.5×10^4 Bohr magnetons, respectively. The sizes of the particles are also mentioned in the insets. The error bars are the deviations from the fits.

9. Temperature estimation

Simulations of scattering and heat transfer were carried out to calculate the absorbed power and the temperature variation of the nanoparticle, respectively. The nanoparticle scattering model is shown in Figure S14 (a). Considering the symmetry of this model, we only take a quarter of the whole model to reduce the calculation time. The particle is at the center of the simulated volume and surrounded by the refractive medium. The mismatch of refractive index between substrate and medium has been ignored. Plane circularly polarized heating and probe beams are taken as background fields. The focusing configurations are different for these two beams, the focus beam waist of the heating beam is 3 μm and that of the probe beam is 656 nm (1.22 λ/NA). The heating and probe powers absorbed by the particle can be obtained by a far-field scattering calculation. To get the temperature of the nanoparticle, we calculate heat diffusion from the nanoparticle to the substrate and medium. Here, we considered the different thermal conductivities of glass and hexadecane. The model and boundary conditions are shown in Figure S14 (b). The spherical particle is located on the surface of the glass and surrounded by the liquid. Different heat conductivities have been assigned to the glass and to the liquid medium. The particle is set as a point heat source with the power obtained from the scattering simulation. The outside of this model is maintained at ambient temperature (293.15 K). The optical and thermal parameters of materials are shown in Table 1.

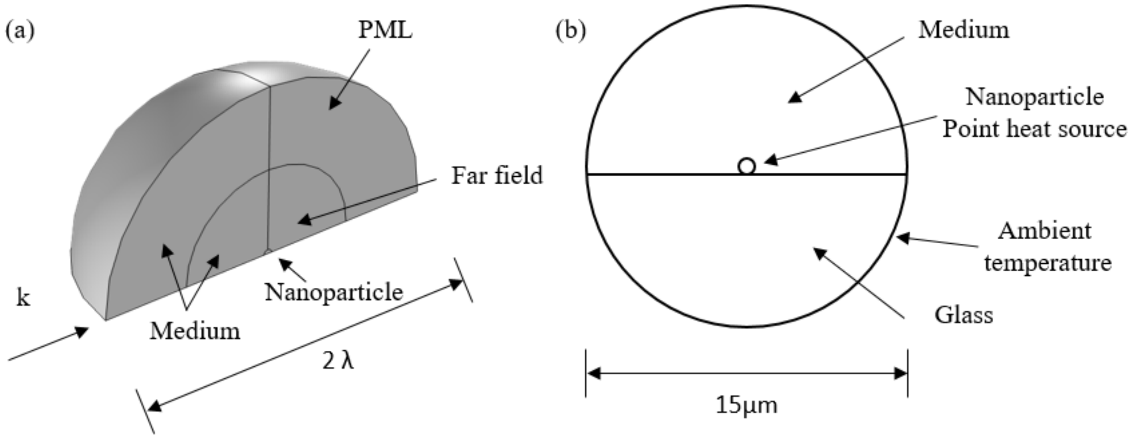

Figure S14: COMSOL model used for the temperature calculation. See details in the text.

	k (W/(m·K))	C_p (J/(kg·K))	ρ (kg/m^3)	n
magnetite	6	890	5180	2.13 + i × 0.86 [3]
glass	1.114	860	2510	1.516
Oil	0.14	1972.5	870	1.518
hexadecane	0.14	2215.1	770	1.433

Table 1: Parameters used for the COMSOL simulation to calculate the temperature for magnetite nanoparticles in two photothermal media, oil and hexadecane.

10. Temperature dependence of the switching events of particle P1

As we vary the particle temperature through variable heating and probe powers, we need the absorption coefficients of magnetite at these two wavelengths to estimate the particle temperature. A number of literature data can be found for the refractive indices of magnetite. Here we selected two values from the works of Huffman et al.[4] and that of Triaud et al.[3] Using the COMSOL model described above, we calculated the temperatures and deduced the residence times in up- and down-states shown in Figure S15. Arrhenius fits to both of these temperature dependences show a similar anisotropy energy barrier. In the main text, we chose the refractive index database of Triaud et al. Note that the temperature change due to the modulation between left- and right-handed polarization is in all cases less than 5 K, much lower than the changes induced in these experiments.

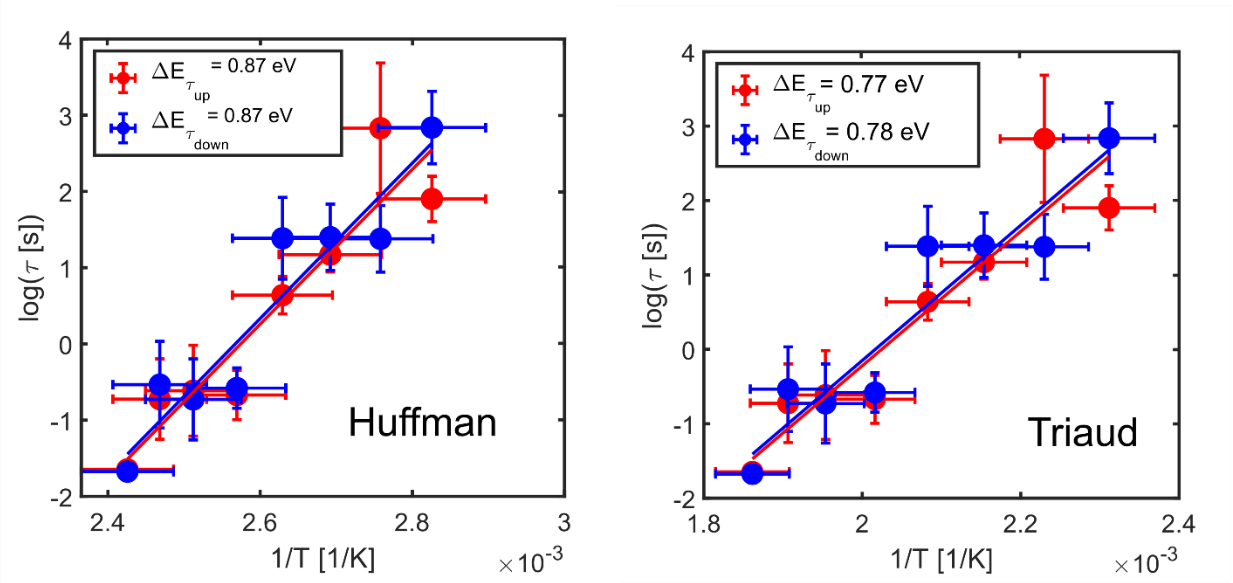

Figure S15: Temperature dependence of residence times in the two magnetization states of particle P1 fitted with the Stoner-Wohlfarth model. The temperature is calculated on the basis of two literature values of the optical absorption of magnetite, by Huffman[4] and Triaud.[3] Both of these temperature dependences yield similar values for the anisotropy energy barrier.

11. Dynamical heterogeneity of a particle measured over several hours

To improve the characterization of the dynamical heterogeneity of magnetic switching, we followed a single magnetite nanoparticle (particle A1) for several hours, but not continuously. We allowed for several waiting times without laser exposure. The results are shown in Figure S16. The particle shows various magnetic behaviors over this longer time period. The mechanism behind the transitions between these behaviors is not well understood. We speculate

that composition fluctuations due to possibly reversible (photo-)oxidation events and/or structural or surface defects and/or PVP ligands on the surface may influence the topography of the magnetic energy landscape, as seen for example in the simulations by Winklhofer et al.[5]

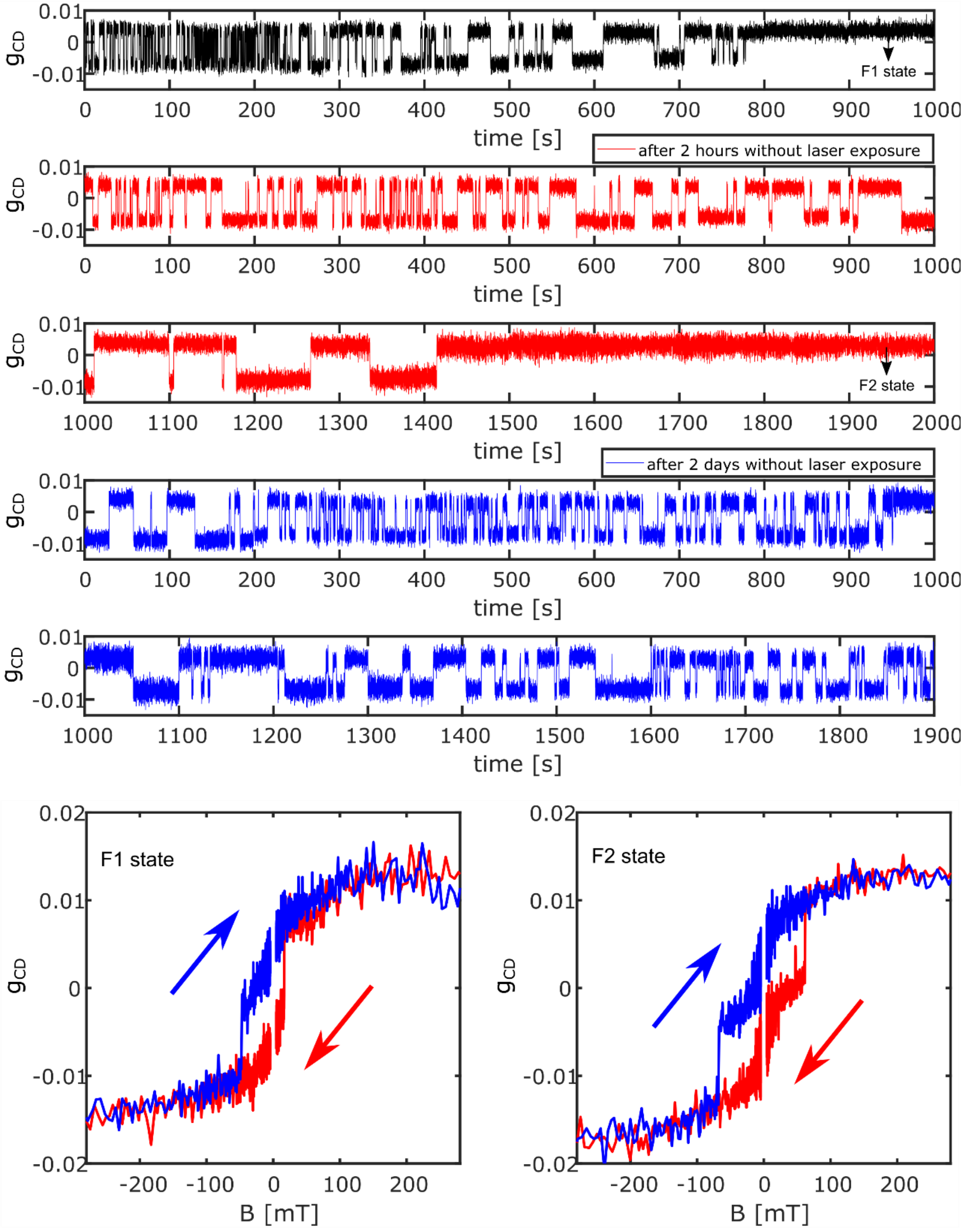

Figure S16: Dynamical heterogeneity in the switching behavior of a single particle (A1) measured over several hours. The black time trace shows dynamical heterogeneity over a

continuous measurement of 1000 s. The intermediate switching behavior slowly becomes ferromagnetic (F1 state) after 800 s, as evidenced by the hysteresis of the magnetization curve measured in the F1 state (bottom left). After 2 hours without laser exposure, the particle again resumes switching, as shown by the red time trace. Over the next period of 2000 s, the particle reverts to the ferromagnetic (F2) state, as evidenced by the hysteresis loop in the magnetization curve measurement of the F2 state (bottom right). After 2 days without laser exposure, the particle resumes switching again and remains in the intermediate switching state for over 1900 s. Arrows indicate the direction of magnetic field sweep.

12. Dynamical heterogeneity of UV-plasma-cleaned particles

To check if the surface ligands alone are responsible for the dynamical heterogeneity, we cleaned the magnetite nanoparticles in UV-plasma. Switching measurements of such particles (particle B1 and particle B2) are shown in Figure S17. These particles still show pronounced dynamical heterogeneity. Therefore, we conclude that surface ligands cannot be the only cause of dynamical heterogeneity.

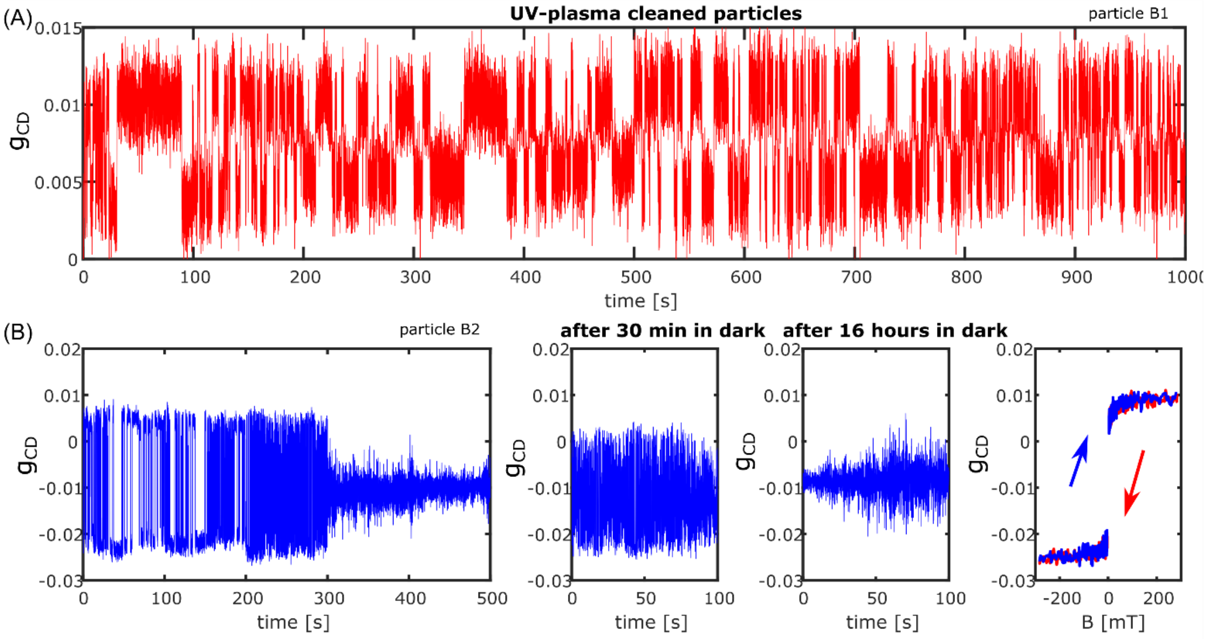

Figure S17: Dynamical heterogeneity in the switching behavior of two particles (particle B1 and particle B2) which were UV-plasma cleaned. (A): Particle B1 shows a change of switching rates over time but still remains in the intermediate magnetic state. (B): Particle B2 shows a change from the intermediate switching magnetic behavior to a quickly switching, superparamagnetic state. Without laser exposure for 30 min, the particle seems to slow somewhat in its switching rate, but still remains very fast compared to the earlier intermediate state observed at the beginning of the trace. Here, 16 hours without laser exposure did not change much the superparamagnetic behavior, as evidenced by the hysteresis-free magnetization curve. Arrows indicate the direction of magnetic field sweep.

13. Dynamical heterogeneity of a particle covered with HfO_2

To check whether surface oxidation influences the dynamical heterogeneity behavior of our magnetite nanoparticles, we deposited a thin (5 nm) layer of HfO_2 by ALD (atomic layer deposition) which would protect the sample from oxidation. We measured two particles (particle C1 and particle C2) as shown in Figure S18. Both these particles show changes of switching rates over time, and in both cases, the switching rate becomes faster over time. In the case of particle C2, we observed three states at the beginning of the trace, which later changed to two states, upon disappearance of one of the states, another possible manifestation of dynamical heterogeneity. The appearance of three states in particle C2 is not understood, and is possibly due to crystalline anisotropy and shape anisotropy both influencing the magnetic energy landscape of particle C2.

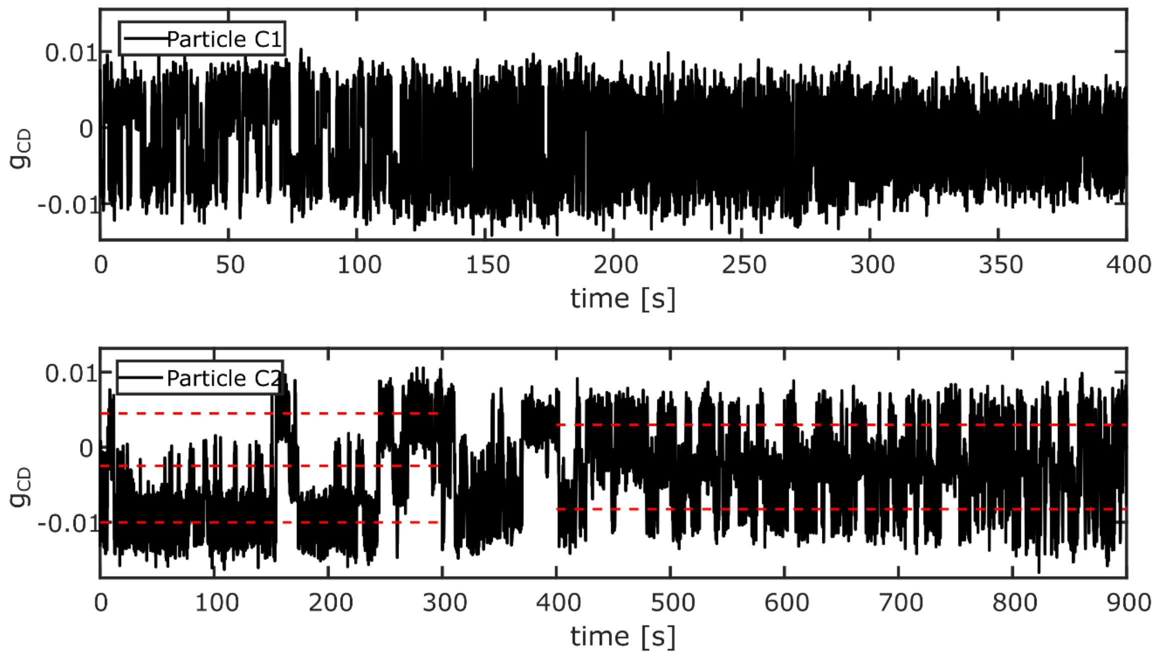

Figure S18: Dynamical heterogeneity of the switching of two particles (particle C1 and particle C2) measured in a magnetite nanoparticle sample coated by about 5 nm of HfO_2. Both these particles show changes of switching rates over time i.e., dynamical heterogeneity.

14. Dynamical heterogeneity of a particle which was plasma-cleaned and covered with HfO_2

To avoid the effects of both surface oxidation and ligands on the dynamical heterogeneity, we prepared a sample which was plasma-cleaned and subsequently covered with 5 nm HfO_2. We measured time traces of three particles (particle D1, particle D2 and particle D3) as shown in Figure S19. Although particle D2 shows weak dynamical heterogeneity, the other two particles show quite pronounced dynamical heterogeneity. This result suggests that the changes which

induce dynamical heterogeneity are probably taking place inside the particle. Internal charge reordering events upon heating have previously been reported.[6]

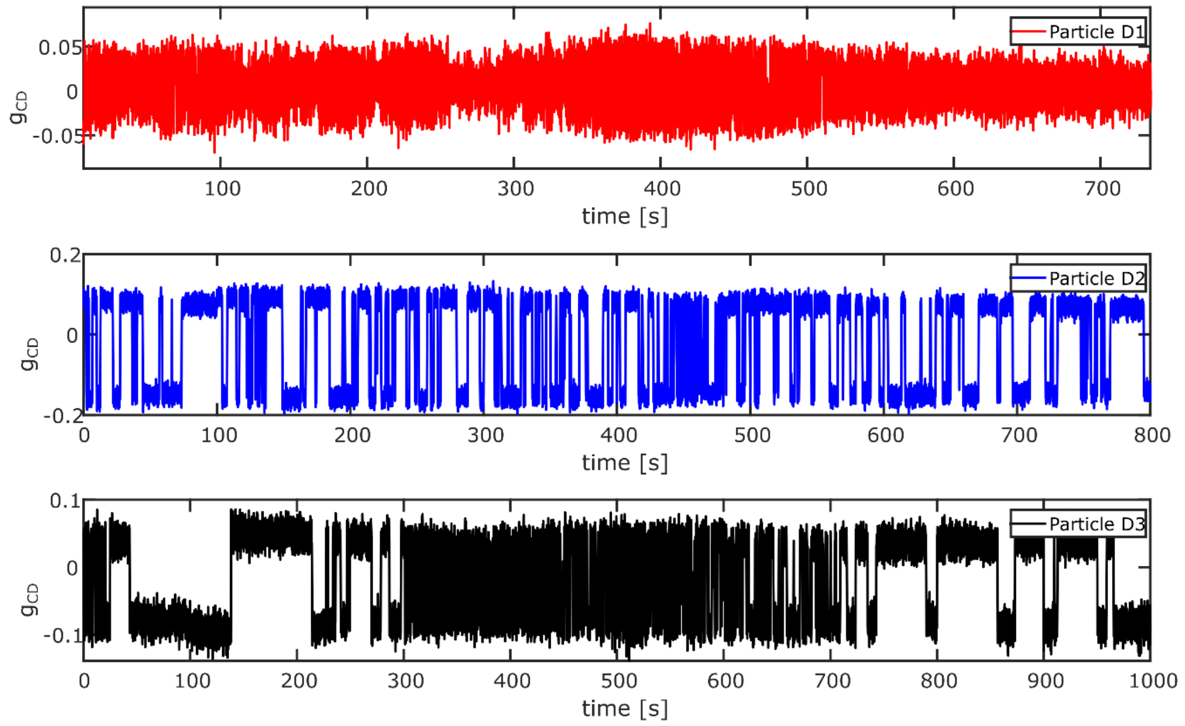

Figure S19: Time traces of three particles (particle D1, particle D2 and particle D3) measured in a plasma-cleaned sample covered with 5 nm HfO_2. Time traces showing the dynamical heterogeneity behavior.

15. Discussion of the terminology of the g-factor and the dissymmetry factor g_{CD}

In the field of single-particle circular dichroism spectroscopy, the so-called dissymmetry factor i.e., the differential absorption of left and right circularly polarized light (i.e., CD) normalized by total absorption is traditionally called "g-factor". To avoid confusion with the Landé factor *g* in magnetism, well-known in the calculations of magnetic moments and angular momentum states of atoms, we referred the CD g-factor as "g_{CD}". We recommend the use of this terminology in future work.

16. Estimation of inter-particle magnetic field in our sample

When particles are close-by, the dipolar field of nearby particles can influence a particle's magnetic properties. If the radius of the particle is R and the distance of the particle from the near-by particle is r, the mean field produced by the near-by particle is $(R/r)^3 \times B_s$, where B_s is the field at the particle surface. For our case, for example particle P1 in the main text (see Figs. 1E and 2A), B_s is above 10 mT, R is about 20 nm and r is more than 1 μm. Therefore, the mean field produced by a particle at a distance of 1 μm on particle P1 would be about 80 nT. Due to such a

low value, we neglected the mean field created by other particles in the magnetization switching analysis.

17. Magnetic field vs distance

Figure S20 shows the magnetic field measured with a Gaussmeter upon varying the distance between the Hall probe and the magnet. A typical field required to saturate a particle like P1 in the main text is below 20 mT at which value the external magnet is more than one cm away from the sample. At such a distance, we assumed the magnetic field to be uniform. However, the magnetic field near the sample could be more heterogeneous, which could introduce some inaccuracy on the largest field values.

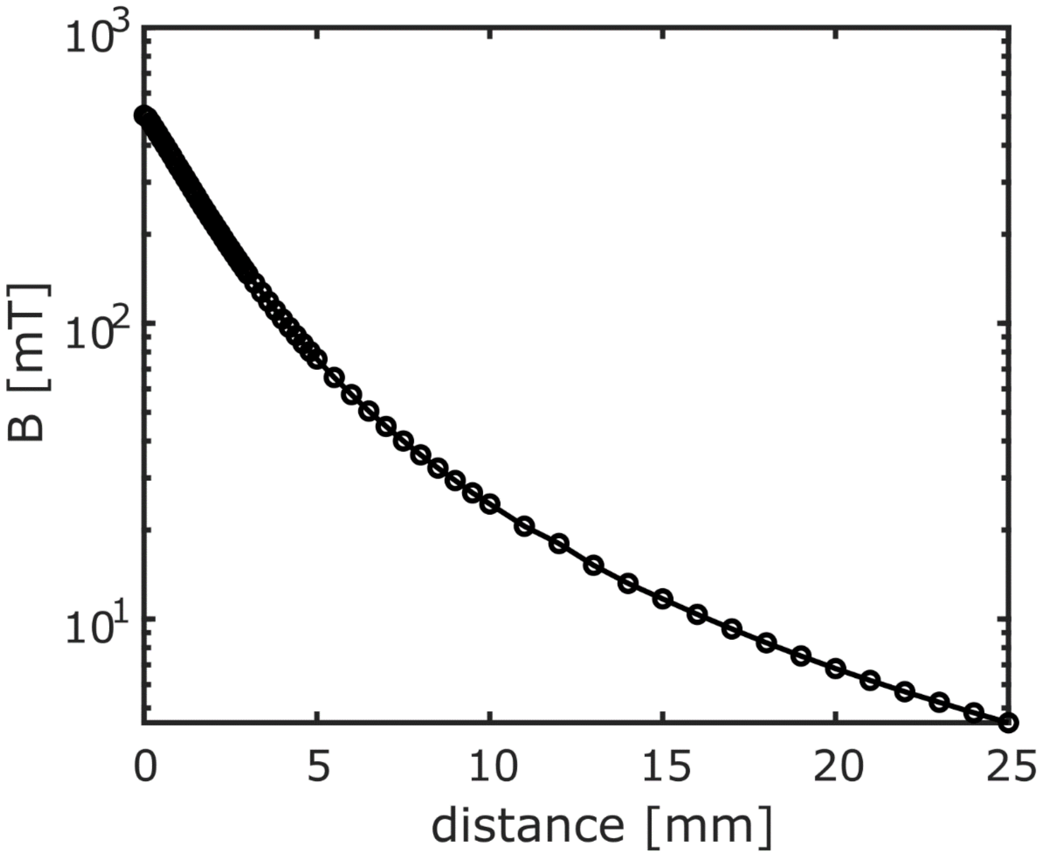

Figure S20: Plot of the magnetic field measured with a Gaussmeter vs the distance between the magnet and the Hall probe.

18. Basic principle of the PT MCD technique

Details about the PT MCD method can be found in our previous paper[7]. Here we briefly recall the basic principles of the method. The PT MCD method is based on photothermal microscopy. When an absorbing nanoobject is illuminated with light (which we call the heating beam), the nanoparticle creates heat through non-radiative relaxation and dissipates it into its surroundings.

The heat dissipation creates a temperature profile (which is an *1/r* profile in steady state; *r* is the distance from the particle). Due to the thermo-refractive behavior of the surrounding medium, the temperature profile creates a refractive index profile which is called the thermal lens. A second beam (which we call the probe beam) is used to probe the thermal lens. The scattered probe light interferes with the incident probe beam (either reflected or transmitted). A modulated interference signal due to modulation of the heating laser is filtered using a sensitive lock-in amplifier, which provides the photothermal signal. Therefore, the photothermal signal gives information about the absorption of the nanoobject. This basic principle provides the contrast of photothermal microscopy.

Circular dichroism (CD) is defined as the differential absorption of left and right circularly polarized light. It is a specific property of chiral nanoobjects. When a chiral nanoparticle is heated by absorption of a beam with polarization modulated between left- and right-circular states, the chiral nanoparticle produces a modulated photothermal signal which is called photothermal circular dichroism (PT CD).

Photothermal magnetic circular dichroism (PT MCD) is the differential absorption signal of a magnetic nanoparticle in the presence of an external magnetic field. It is due to the polar magneto-optical Kerr (MOKE) effect. The magnetic field is applied along the optical axis. With the flip of magnetic field direction, the magnetic moment orients along the magnetic field and thus a signature of PT MCD is the flip of the sign with flip of the field direction. The strength of the PT MCD signal is proportional to the magnetic moment of the nanoparticle and the so-called g_{CD} factor is proportional to the magnetization of the particle.

19. Detection sensitivity

The particle P1 in the main text has a magnetic moment of 4×10^5 Bohr magnetons. As shown in Fig. 1C, the signal-to-background of MCD signal is more than 10 with an integration time of 100 ms. Therefore, the detection sensitivity of our method on that time scale is better than 4×10^4 Bohr magnetons. As an example, the particle P8 shown in Figs. S12-13 has a magnetic moment of 0.95×10^5 Bohr magnetons which is about 2.5 times the detection sensitivity. We have measured the magnetization switching of this particle and the magnetic moment is calculated from the fit according to the Stoner-Wohlfarth model. The detection sensitivity can still be improved with an increase in integration time.

20. Possible applications of PT MCD

PT MCD can be applied to study magnetization switching under the influence of applied external perturbations such as electric fields, microwave or optical pulses. As a relatively large sample area of many single nanoparticles can be influenced simultaneously by those perturbations, extended statistics of single-particle switching could be established in a relatively short time with a high throughput. More specifically, the switching mechanism could be investigated in each of these cases, either by comparing several repeated switching events of the same particle, or by observing switching events of several different particles and correlating them to their individual properties such as magnetization anisotropy and easy-axis orientation. The influence of external parameters (temperature, electric and magnetic fields, chemical reactions, etc.) could be mapped

on a single-particle basis and correlated with other properties, such as is currently done for single biomolecules.

PT-MCD can also be applied to many magnetic nanomaterials, such as anti-ferromagnetic nanoplatelets[8] and metal-insulator-metal devices[9]. Single-particle studies will remove ensemble-averaging and expose correlations between particle structure and their magnetic properties. Such a study can help us to investigate domain wall propagation and nucleation in magnetic materials at the single-particle level.

21. Calculation of the critical radius for a single-domain magnetite particle

We calculated the critical radius ($r_{critical}$) for a single domain magnetite nanoparticle using the following formula

$$r_{critical} = \frac{36\sqrt{AK}}{\mu_0 M_s^2}$$

where, $A = 10^{-11}$ J/m, $K = 10^4$ J/m^3, $\mu_0 = 1.25 \times 10^{-6}$ N/A^2 and $M_s = 446 \times 10^3$ A/m. The calculated critical radius is about 45 nm which is in good agreement with the data available from the literature[10].

22. Time traces of MCD signals for particles P1 to P6 as labelled in Fig. 1 in the main text

Figure S21 shows time traces of the MCD signal of the six particles labelled in the main text (Fig. 1) P1 to P6. The time traces indicate that particle P1 behaves differently from the other particles. Particle P1 shows intermediate magnetization behavior that displays switching of the MCD signal between two values.

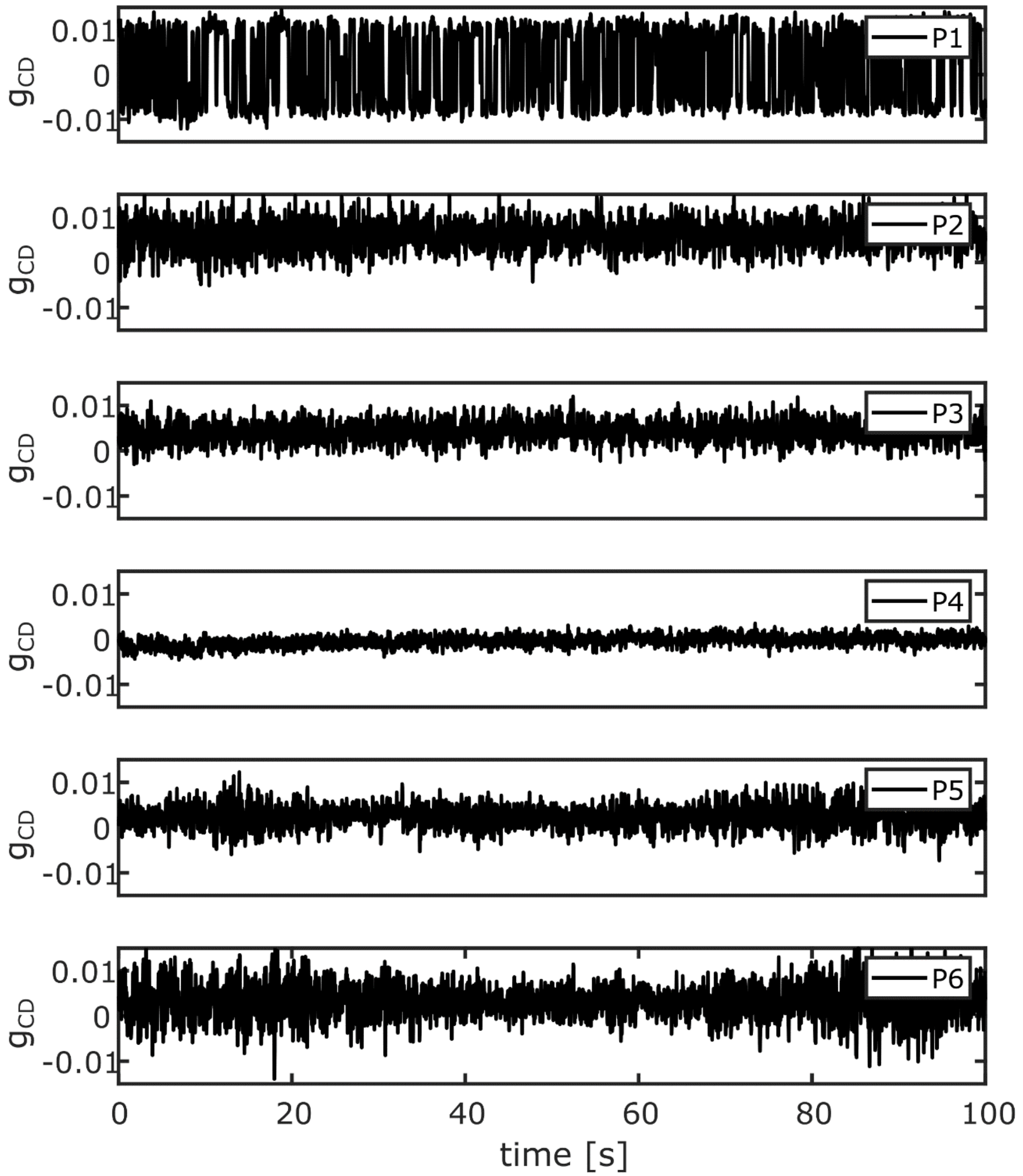

Figure S21: Time traces of MCD signal over 100 s for particles P1-6 from Fig. 1 in the main text.

23. Attempt frequency obtained from the Arrhenius fit of the temperature-dependent switching of Particle P1 in the main text

According to Néel-Brown theory, the lifetime of the up or down state of magnetization switching follows an Arrhenius dependence,

$$\tau = \frac{1}{f_0} exp\left(\frac{\Delta E}{k_B T}\right)$$

where τ is the lifetime of a magnetization state, f_0 is the attempt frequency, ΔE is the energy barrier, k_B is the Boltzmann constant and T is the absolute temperature. In most studies, the attempt frequency[11] is considered as a constant factor in the order of 10^{10} Hz[12]. Krause et al. showed that the attempt frequency depends on the morphology of the particles[13]. In our case, we obtained the attempt frequency from the Arrhenius fit as shown in Fig. 2c in the main text and its value is about 10^8 Hz, which is close to the reported value in the reference[14]. It would be interesting to study size- and shape-dependent magnetization switching using the PT MCD technique; however, this is beyond the scope of the current study.

24. Magnetization curve of a ferromagnetic particle

Figure S22 shows magnetization curves of a ferromagnetic particle calculated using the Stoner-Wohlfarth model (detailed discussion in the section “Stoner-Wohlfarth model”). The magnetization curve is calculated at different angles of the easy axis with respect to the direction of the applied magnetic field. The magnetization curve with the easy axis angle of 90° i.e., the particle’s easy axis along the sample plane, looks similar to the magnetization curve of a superparamagnetic particle with similar easy axis orientation (see Figure S4). Therefore, it is difficult to distinguish between superparamagnetic and ferromagnetic behavior from the magnetization curve when the particle’s easy axis is perpendicular to the applied magnetic field.

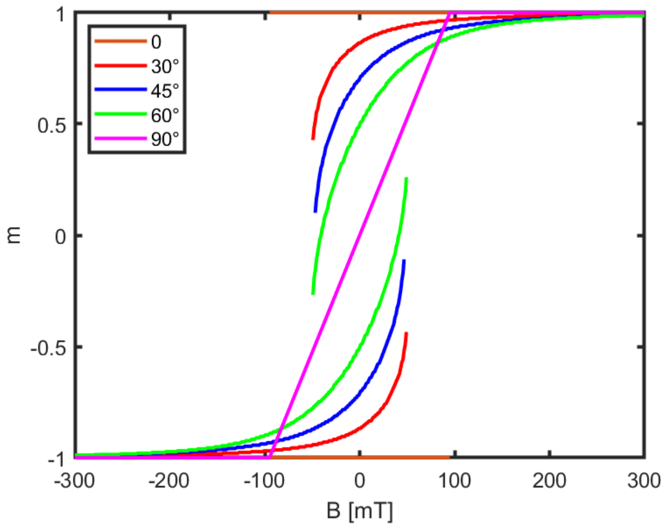

Figure S22: Magnetization curve of a ferromagnetic particle (aspect ratio of 1.5) calculated using the Stoner-Wohlfarth model. The angles mentioned in the inset are the angle of the easy axis with respect to the direction of the external field.

25. Analysis of dynamical heterogeneity of another time trace similar to that of Fig. 3

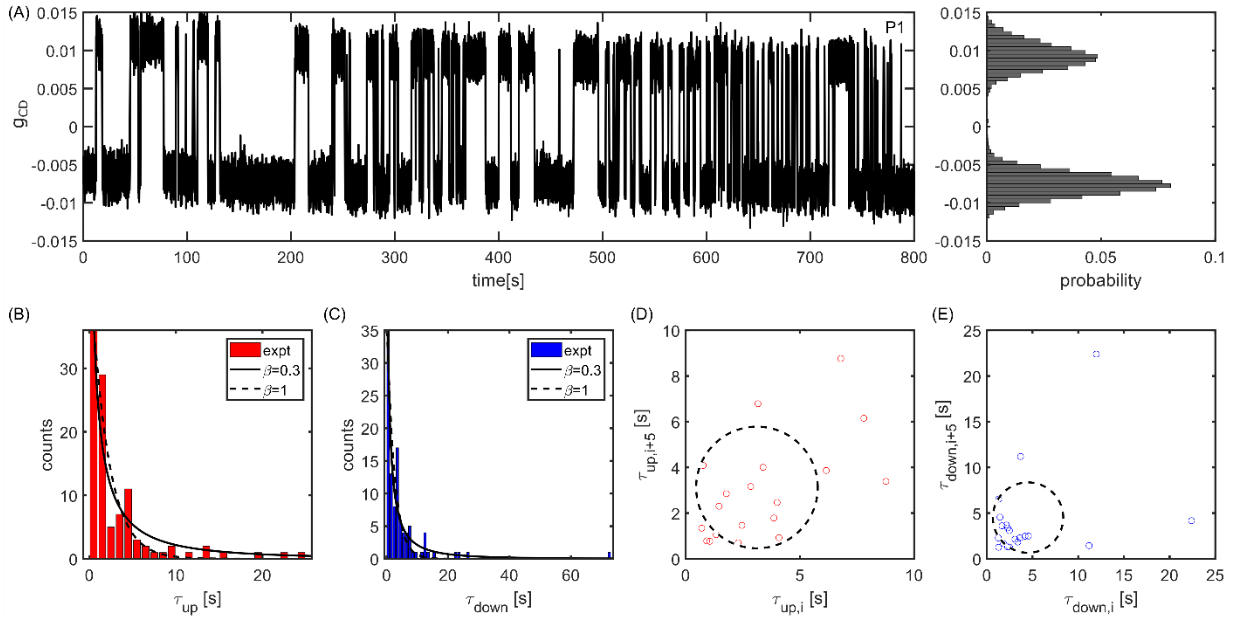

Figure S23: Dynamical heterogeneity of magnetization switching of particle P1. A: Time trace of magnetization switching over 800 s. The corresponding histogram of g_{CD} is shown on the right. B, C: Histograms of τ_{up} and τ_{down} with stretched-exponential fits (stretching exponents β given in insets). D, E: Correlation plots of successive averages of τ_{up} and τ_{down}, averaged over five successive events. Strong fluctuations of these averages lead to correlation points out of the dashed discs (see details in the main text).

26. Size distribution of single magnetite nanoparticles

The photothermal signal of a single small particle is proportional to its volume and to its Clausius-Mossotti factor. With a particle of a known size and material, one can calibrate the photothermal signal to determine the size of a particle of a different material. We used 10 nm gold nanoparticles to calibrate the photothermal signal of magnetite nanoparticles to obtain their sizes, with the known refractive indices of magnetite. We found two sets of data for the magnetite refractive index, as mentioned earlier in the text. For both data sets, the size distribution obtained by the above-mentioned calibration matches the size distribution obtained

from the TEM measurement reasonably well. However, the data of Triaud et al. [3] give a better agreement than those of Huffman et al. [4], as shown in Figure S24.

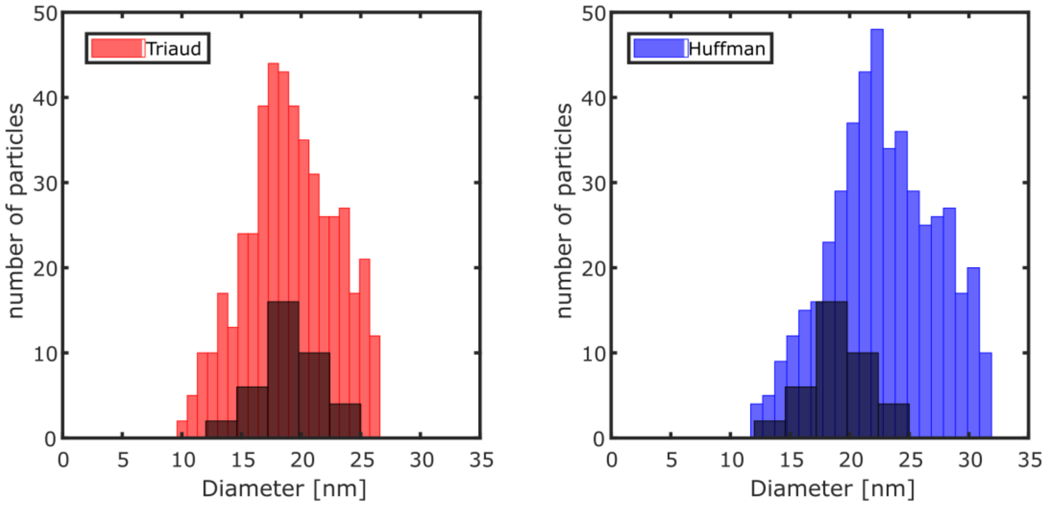

Figure S24: The size distribution of single magnetite nanoparticles obtained from their photothermal signals as shown in Figure S1, using two refractive index databases; (a) Triaud and (b) Huffman as mentioned earlier in the text. The size distributions are compared to the size distribution obtained from TEM as shown in Figure S2.

27. <u>Absorption spectra of magnetite</u>:

Figure S25 shows the absorption spectra of 20 nm magnetite nanoparticles according to the manufacturer (Nanocomposix).

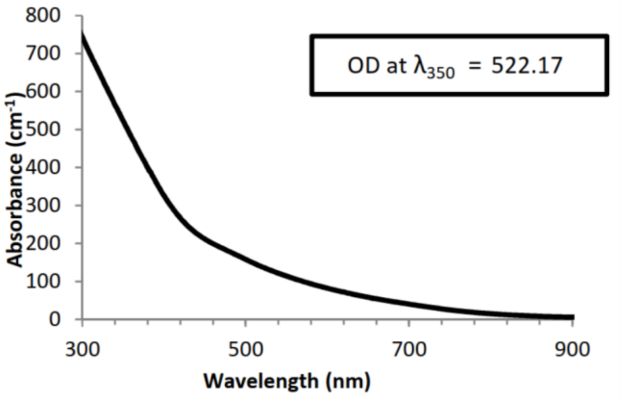

Figure S25: Absorption spectra of magnetite provided by the manufacturer.

Sample preparation

Magnetite (Fe_3O_4) nanoparticles 20 nm in diameter, coated with polyvinylpyrrolidone (PVP) were purchased from Nanocomposix (product number: MGPB20). Hexadecane was purchased

from Sigma-Aldrich (product number: H6703). The stock solution of the magnetite nanoparticles was diluted 1,000 times in an aqueous solution. 15 µl diluted solution was spin-coated on a UV-plasma-cleaned glass coverslip (thickness about 170 µm) at 2000 rpm for 30 s to disperse the particles homogeneously on the glass surface. The glass coverslip was sandwiched with a cavity glass slide with a thickness of about 1.4 mm which contained a cavity to hold the liquid used for the photothermal measurement, hexadecane. All the measurements mentioned in the main text were done on magnetite nanoparticles immersed in hexadecane. In some measurements reported in the present supplementary section, magnetite nanoparticles were measured in immersion oil. In that case, the same immersion oil used for the microscope objective was used as the photothermal medium.

Optical setup

The details of the optical setup are described in our recent publication.[22] Here we give a brief description of the setup (schematic of the setup is shown in Fig. S26) and the modifications which have been performed to increase the sensitivity to measure 20 nm magnetite nanoparticles. The heating laser (wavelength of 532 nm) was passed through an electro-optical modulator (EOM) and a photo-elastic modulator (PEM) which modulated the laser's polarization at frequencies of 33.5 kHz and 50 kHz, respectively. The dual modulation of the laser polarization created a circular dichroism (CD) signal at the sum frequency as discussed in our previous publication[26]. The heating laser was focused in the back-focal plane of the immersion-oil objective (NA= 1.45) and illuminated the sample in a wide-field area of about 3 µm diameter at half maximum. Such smaller illumination area allowed higher intensity; however, it required very careful polarization control which had been regularly checked during each experiment using a calibration method proposed in our previous publication.[22] The collimated circularly polarized continuous-wave probe beam of wavelength 780 nm was focused at the sample using the same objective. The scattered probe beam was detected in the reflection mode, was filtered from the heating beam using a band-pass filter (BP780) and focused on a photodiode using a lens (focal length 75 mm). The CD signal at the sum frequency, 83.5 kHz was detected using a lock-in amplifier. To vary the magnetic field, a long permanent cylindrical NdFeB magnet (a set of small cylindrical magnets of diameter 3 mm) was placed perpendicular to the sample and its position was varied to change the field. To invert the direction of the magnetic field, the magnetic poles were inverted.

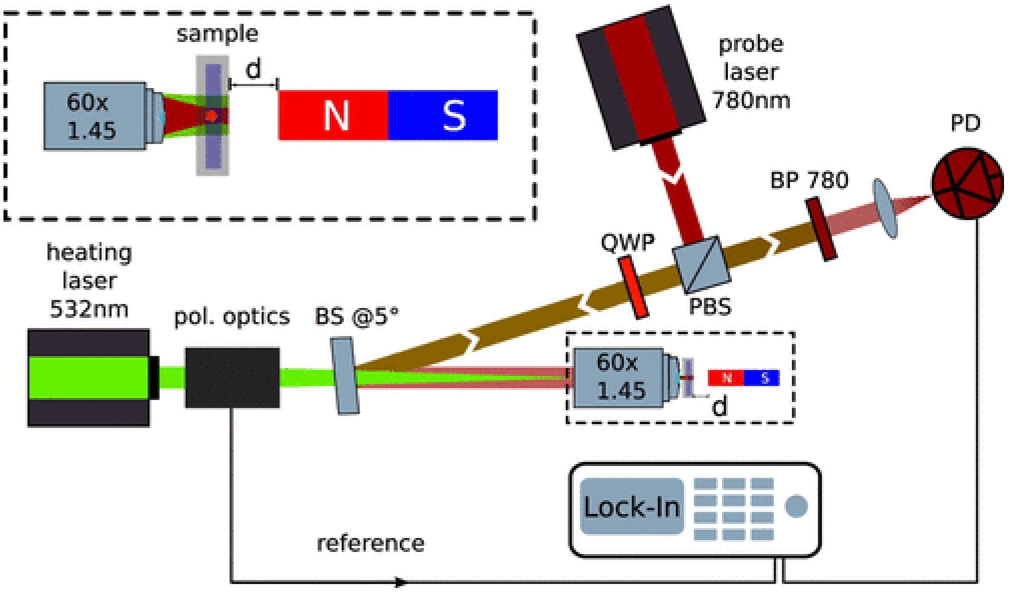

Figure S26: The figure is taken from our previous publication[7]. Heating laser of 532 nm wavelength is passed through a combination of polarization optics to modulate light between left and right circular polarizations. Heating laser is focused on the back-focal plane of the objective (60× magnification and NA of 1.45) to create a Koehler illumination. The probe laser of 780 nm is passed through a combination of polarizing beam splitter (PBS) and quarter-wave plate (QWP) to create circular polarization and then focused on the sample with the high NA objective after reflecting at the 50/50 beam splitter (BS). Photothermal signal is detected in the backward detection using a photodiode (PD) which is connected to a lock-in amplifier. A 780 nm band-pass (BP) filter is used to filter out the heating laser. To measure magnetic circular dichroism signal, an external magnet is used. To vary the magnetic field, the distance between the sample and the magnet is varied and to flip the magnetic field direction, the magnet's orientation is flipped.

References:

(1) Moreno, R.; Poyser, S.; Meilak, D.; Meo, A.; Jenkins, S.; Lazarov, V. K.; Vallejo-Fernandez, G.; Majetich, S.; Evans, R. F. L. The Role of Faceting and Elongation on the Magnetic Anisotropy of Magnetite Fe3O4 Nanocrystals. *Sci Rep* **2020**, *10* (1), 2722. https://doi.org/10.1038/s41598-020-58976-7.

(2) Respaud, M. Magnetization Process of Noninteracting Ferromagnetic Cobalt Nanoparticles in the Superparamagnetic Regime: Deviation from Langevin Law. *Journal of Applied Physics* **1999**, *86* (1), 556–561. https://doi.org/10.1063/1.370765.

(3) Roush, T. L.; Teodoro, L. F. A.; Blewett, D. T.; Cahill, J. T. S. Optical Constants and Diffuse Reflectance of Opaque Minerals: A Modeling Study Using Magnetite. *Icarus* **2021**, *361*, 114331. https://doi.org/10.1016/j.icarus.2021.114331.

(4) Huffman, D. R. Interstellar Grains The Interaction of Light with a Small-Particle System. *Advances in Physics* **1977**, *26* (2), 129–230. https://doi.org/10.1080/00018737700101373.

(5) Winklhofer, M.; Fabian, K.; Heider, F. Magnetic Blocking Temperatures of Magnetite Calculated with a Three-Dimensional Micromagnetic Model. *Journal of Geophysical Research: Solid Earth* **1997**, *102* (B10), 22695–22709. https://doi.org/10.1029/97JB01730.
(6) Elnaggar, H.; Graas, S.; Lafuerza, S.; Detlefs, B.; Tabiś, W.; Gala, M. A.; Ismail, A.; van der Eerden, A.; Sikora, M.; Honig, J. M.; Glatzel, P.; de Groot, F. Temperature-Driven Self-Doping in Magnetite. *Phys. Rev. Lett.* **2021**, *127* (18), 186402. https://doi.org/10.1103/PhysRevLett.127.186402.
(7) Spaeth, P.; Adhikari, S.; Lahabi, K.; Baaske, M. D.; Wang, Y.; Orrit, M. Imaging the Magnetization of Single Magnetite Nanoparticle Clusters via Photothermal Circular Dichroism. *Nano Lett.* **2022**. https://doi.org/10.1021/acs.nanolett.2c00178.
(8) Li, J.; van Nieuwkerk, P.; Verschuuren, M. A.; Koopmans, B.; Lavrijsen, R. Substrate Conformal Imprint Fabrication Process of Synthetic Antiferromagnetic Nanoplatelets. *arXiv:2206.15320v1* **2022**.
(9) Li, X.; Zanotti, T.; Wang, T.; Zhu, K.; Puglisi, F. M.; Lanza, M. Random Telegraph Noise in Metal-Oxide Memristors for True Random Number Generators: A Materials Study. *Advanced Functional Materials* **2021**, *31* (27), 2102172. https://doi.org/10.1002/adfm.202102172.
(10) Reichel, V.; Kovács, A.; Kumari, M.; Bereczk-Tompa, É.; Schneck, E.; Diehle, P.; Pósfai, M.; Hirt, A. M.; Duchamp, M.; Dunin-Borkowski, R. E.; Faivre, D. Single Crystalline Superstructured Stable Single Domain Magnetite Nanoparticles. *Sci Rep* **2017**, *7*, 45484. https://doi.org/10.1038/srep45484.
(11) Kagan, Yu. Quantum Diffusion in Solids. *J Low Temp Phys* **1992**, *87* (3), 525–569. https://doi.org/10.1007/BF00114916.
(12) Brown, W. F. Thermal Fluctuations of a Single-Domain Particle. *Phys. Rev.* **1963**, *130* (5), 1677–1686. https://doi.org/10.1103/PhysRev.130.1677.
(13) Krause, S.; Herzog, G.; Stapelfeldt, T.; Berbil-Bautista, L.; Bode, M.; Vedmedenko, E. Y.; Wiesendanger, R. Magnetization Reversal of Nanoscale Islands: How Size and Shape Affect the Arrhenius Prefactor. *Phys. Rev. Lett.* **2009**, *103* (12), 127202. https://doi.org/10.1103/PhysRevLett.103.127202.
(14) Mamiya, H.; Fukumoto, H.; Cuya Huaman, J. L.; Suzuki, K.; Miyamura, H.; Balachandran, J. Estimation of Magnetic Anisotropy of Individual Magnetite Nanoparticles for Magnetic Hyperthermia. *ACS Nano* **2020**, *14* (7), 8421–8432. https://doi.org/10.1021/acsnano.0c02521.
